# Qualitative and Quantitative Secondary Metabolite Profiles in a Large Set of Sumatra Benzoin Samples

**DOI:** 10.1021/acs.jafc.3c01861

**Published:** 2023-07-03

**Authors:** Ming Yuan Heng, Nova Syafni, Justine Ramseyer, Barbara Thuerig, Lucius Tamm, Matthias Hamburger, Olivier Potterat

**Affiliations:** † Pharmaceutical Biology, University of Basel, Basel CH-4056, Switzerland; ‡ Faculty of Pharmacy and Sumatran Biota Laboratory, Andalas University, Kampus Limau Manis, Padang, West Sumatra 25163, Indonesia; § Research Institute of Organic Agriculture FiBL, Ackerstrasse 113, Frick CH-5070, Switzerland

**Keywords:** Sumatra benzoin, Styrax benzoin, Styrax paralleloneurum, *p*-coumaryl cinnamate, sumaresinolic acid, phytochemical profile

## Abstract

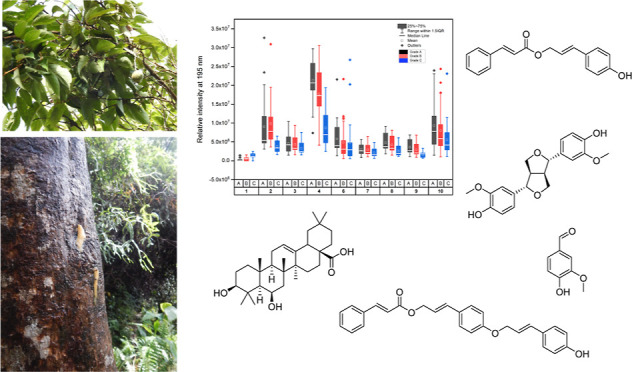

Sumatra benzoin, a resin produced by *Styrax benzoin* and *Styrax paralleloneurum*, is used as an aromatic agent and may have the potential to be developed as a new agricultural fungicide. In this context, we performed a comprehensive metabolite profiling of a commercial grade A resin by high-performance liquid chromatography coupled with photodiode array detection, evaporative light scattering detection, and mass spectrometry (HPLC-PDA-ELSD-MS) analysis in combination with ^1^H NMR. Thirteen compounds including a new cinnamic acid ester containing two *p*-coumaroyl residues were identified after preparative isolation. These compounds accounted for an estimated 90% of the crude resin according to ^1^H NMR analysis. The two major constituents, *p*-coumaryl cinnamate (**5**) and sumaresinolic acid (**11**), were quantified by HPLC analysis. In a next step, the chemical profiles and the content in *p*-coumaryl cinnamate were compared in a large set of resin samples of different quality grades that were obtained from various commercial suppliers in Sumatra. The qualitative profiles of the samples were very similar, but significant quantitative differences were observed between different quality grades and origins of the samples for the relative contents.

## Introduction

Benzoins are balsamic resins produced by several trees of the genus *Styrax* belonging to the family Styracaceae. Two commercially valuable benzoins are listed in the European Pharmacopoeia.^1^ Siam benzoin is produced by *Styrax tonkinensis* (Pierre) Craib ex Hartwich growing in Thailand, Laos, Vietnam, and Cambodia. Sumatra benzoin is obtained from *Styrax paralleloneurum* Perkins and *Styrax benzoin* Dryander found mainly in the island of Sumatra, Indonesia. The resins are collected after making incisions in the bark similar to rubber tapping. Benzoins have been used in perfumes, incenses, and in medicines since ancient times.^2^ Nowadays, benzoins are used as aromatic agents in the production of soaps, in cosmetic preparations, to flavor tobacco, or as a fixative agent in pharmaceuticals.^3^ Sumatra benzoin has the higher market share with a volume of around 8000 tonnes per year and a benzoin forest area covering approx. 23 000 ha.^4^ Siam benzoin consists mainly of coniferyl benzoate, benzoic acid, and triterpenoids including 6β- and 19α-hydroxylated oleanolic acid derivatives such as sumaresinolic acid and siaresinolic acid,^5−8^ while Sumatra benzoin contains pinoresinol, sumaresinolic acid, and large amounts of cinnamic acid and esters such as *p*-coumaryl cinnamate and cinnamyl cinnamate.^8^

Cinnamic acid esters have been reported to exhibit antibacterial and antifungal activity.^9,10^ We recently found that Sumatra benzoin and its major constituent, *p*-coumaryl cinnamate, may have a potential to be developed as an agricultural fungicide.^11^ Several phytochemical investigations have been performed on Sumatra benzoin. They include gas chromatography (GC) analysis of volatile constituents,^12,13^ the detection and quantification of phenolic constituents by high-performance liquid chromatography with ultraviolet-evaporative light scattering detection (HPLC-UV-ELSD) analysis,^8^ the analysis of triterpenoids and benzyl and cinnamyl esters by HPLC coupled with photodiode array detection and fluorimetry (HPLC-PDA-fluorimetry),^14^ and the analysis of free and ester bound benzoic and cinnamic acids by GC–MS and HPLC–frit-fast atom bombardment–MS (HPLC–frit FAB–MS).^15^ Sumatra benzoin was also included in a large study comparing the chemical profiles of various balsams.^16^ However, with the exception of one report on the volatile constituents of 13 Sumatra benzoin gums,^13^ these studies included only a limited number of samples. The qualitative and quantitative profiles of Sumatra benzoin constituents may be influenced by several factors. The resin-producing *Styrax* trees grow in various regions of Sumatra under different environmental conditions. Unique vernacular names in local village dialects may be a confounding factor in the unambiguous identification of benzoin-resin-producing trees.^17^ Factors such as tree management and harvesting protocols may lead to a variable quality of resins. Moving down the supply chain, the quality of the resins is affected by post-harvest processing by merchants, such as drying and pre-grading of resins according to size and visual appearance.^18^

In this context, we performed a comprehensive analysis of the chemical profile of a commercial grade A Sumatra benzoin sample by HPLC coupled with photodiode array detection, evaporative light scattering detection, and mass spectrometry (HPLC-PDA-ELSD-MS) combined with ^1^H NMR analysis of the crude resin. Subsequently, a large set (*n* = 118) of resin samples of different quality grades and origins were analyzed for their content in the main constituent *p*-coumaryl cinnamate. Finally, variations in chemical profiles and in the relative contents of identified metabolites were investigated in a selection of 81 resin samples of various origins and quality grades.

## Materials and Methods

### Plant Material

Sumatra benzoin (reference grade A resin) used for compound isolation, phytochemical profiling, and quantification of sumaresinolic acid was purchased from Alfred Galke GmbH (Gittelde, Germany). A voucher specimen (Nr. 959) is kept at the Division of Pharmaceutical Biology. For analytical comparison, a total of additional 118 samples of various origins in North Sumatra were purchased from UD. Sentral Utama, Magelang, Central Java, Indonesia. The samples came from 20 different providers. From each provider, one to eight lots were analyzed, each of them consisting of two (A, B) or three quality grades (A, B, and C) according to the size of the resin obtained.^3^

### Chemicals

Ethanol (96%) was obtained from Brenntag Schweizerhall AG (Basel, Switzerland). HPLC-grade acetonitrile and methanol were purchased from Avantor Performance Materials (Radnor Township, PA, USA). Technical-grade ethyl acetate and *n*-hexane were from Scharlab S.L. (Barcelona, Spain) and were redistilled before use. Formic acid was purchased from Scharlab. Ultrapure water was obtained from a Milli-Q water purification system (Merck Millipore, Darmstadt, Germany). Silica gel 60 F254 coated aluminum TLC plates and silica gel (0.043–0.063 mm) for flash chromatography were purchased from Merck KGaA (Darmstadt, Germany). NMR solvents were from Armar Isotope, Dottingen, Switzerland (methanol-*d*_4_, chloroform-*d*) and Acros Organics, NJ USA (DMSO-*d*_6_).

### General Experimental Procedures

Flash chromatography was carried out on a Puriflash 4100 system (Interchim, Montluçon, France) connected to a glass column (45 × 8 cm i.d.). Preparative HPLC was carried out on a Preparative LC/MSD System (Agilent Technologies, Santa Clara, CA, USA) consisting of a binary pump (1260 Prep Bin Pump, 1290 Infinity II), a quaternary pump (G1311A Quat Pump, 1200 Series, post-split make-up flow for ESI-MS detection), a 1290 Infinity II Valve Drive manual injection system, a PDA detector (1100 Series), and a Quadrupole LC/MS system (6120). Separations were performed on a SunFire Prep C_18_ OBD column (5 μm, 150 × 30 mm i.d., Waters, Milford, MA, USA) equipped with a C_18_ Prep guard column (10 × 30 mm i.d.). MeCN and water, both containing 0.1% formic acid, were used as mobile phase. The flow rate was 25 mL/min. UV detection at 195 nm was used for peak collection if not stated otherwise. Semi-preparative HPLC separations were performed on an HP 1100 Series system (Agilent Technologies, Santa Clara, CA, USA) consisting of a binary pump (G1312A BinPump), an auto sampler (G1367A WPALS), a column oven (G1316A COLCOM), and a diode array detector (G1315A DAD). Separations were carried out on a ReproSil-Pur 120 C_18_-AQ column (3 μm, 150 × 3.0 mm i.d., Dr. Maisch, Germany). MeCN and water, both containing 0.1% formic acid, were used as mobile phase. The flow rate was 5 mL/min. The detection wavelength was set at 195 nm. Data were recorded and analyzed using Openlab CDS (Rev.C.01) (Agilent Technologies, USA).

High-resolution electrospray ionization mass spectrometry (HRESIMS) spectra were acquired on a Q Exactive HF Orbitrap LC–MS/MS System (Thermo Scientific, MA, USA). Data processing was performed with MS Workbook Suite (Version 2020.1.2) (ACD/Lab, Toronto, Canada). NMR spectra were recorded with a Bruker Avance III spectrometer (Fällanden, Switzerland) operating at 500 MHz for ^1^H and 126 MHz for ^13^C. All spectra were recorded at 23 °C with a 5 mm BBO probe. Data were processed with NMR Workbook (Version 2020.1.2) (ACD/Lab).

### HPLC-PDA-ELSD-ESIMS Analysis

HPLC-PDA-ELSD-ESIMS analysis was performed on a chromatographic system consisting of a degasser, a quaternary pump (LC-20AD), a column oven (CTO-20AC), a PDA detector (SPD-M20A), a triple quadrupole mass spectrometer (LCMS-8030) (all Shimadzu, Kyoto, Japan), and an ELSD 3300 detector (Alltech, Flawil, Switzerland). Separations were carried out on a SunFire C_18_ column (3.5 μm, 150 × 3.0 mm i.d.) equipped with a guard column (10 mm × 3.0 mm i.d.) (Waters, Milford, MA, USA). The mobile phase consisted of water (A) and acetonitrile (B), both containing 0.1% formic acid. A gradient of 5–95% B in 45 min was applied at a flow rate of 0.4 mL/min. PDA detection was set from 190 to 600 nm. MS data were recorded in the range of *m/z* 100–800, in both positive and negative ion modes. The samples were dissolved in methanol at a concentration of 1 mg/mL, and 2 μL was injected. Data were recorded and analyzed with Lab solutions software (V5.97 SP1) (Shimadzu, Kyoto, Japan).

### Isolation

One portion of Sumatra benzoin (16.0 g) was separated by flash chromatography with a gradient of *n*-hexane and ethyl acetate at a flow rate of 22 mL/min: 0–20 min 33% EtOAc, 20–120 min 33–50% EtOAc, 120–165 min 50–100% EtOAc. Fractions were collected at 1 min intervals and combined into 24 fractions (F1–F24) based on TLC analysis. Fraction F5 (300 mg) was purified by preparative HPLC with a gradient of 5–95% MeCN in 45 min to give compounds **2** (*t*_R_ = 15.6 min, 3.0 mg) and **12** (*t*_R_ = 44.2 min, 5 mg). Purification of fraction F7 (250 mg) by the same procedure yielded compounds **1** (*t*_R_ = 12.6 min, 2 mg), **13** (*t*_R_ = 38.2 min, 10 mg), and **11** (*t*_R_ = 41.3 min, 23 mg), and a subfraction which was further separated by semi-preparative HPLC with 53% MeCN afforded compound **6** (*t*_R_ = 18.7 min, 5 mg). F15 (300 mg) was purified by preparative HPLC as described for F5 to yield compound **11** (*t*_R_ = 38.5 min, 155 mg). MS detection (*m/z* 435–440, positive ion mode) was used for peak collection.

A second portion of Sumatra benzoin (50.0 g) was fractionated by flash chromatography using the above-mentioned method into five combined fractions (F1′- F5′). Fraction F1′ (330 mg) was further separated by preparative HPLC with 65% of MeCN to yield compounds **7** (*t*_R_ = 35.8 min, 14 mg), **8** (*t*_R_ = 36.1 min, 66 mg), and **9** (*t*_R_ = 37.6 min, 92 mg). Fraction F3′ (100 mg) was separated by preparative HPLC with 32% MeCN to give compound **3** (*t*_R_ = 19.1 min, 8 mg). A portion of F2′ (800 mg) was separated by preparative HPLC with a gradient of 30–70% MeCN in 30 min to afford compounds **4** (*t*_R_ = 9.1 min, 277 mg) and **5** (*t*_R_ = 25.1 min, 328 mg). A detailed isolation chart is provided as Supporting Information (Figure S1).

#### (*E*)-3-(4-(((*E*)-3-(4-Hydroxyphenyl)allyl)oxy)phenyl)allyl cinnamate (**10**)

Yellow resin; λ_max_ 272 nm and 212 nm; ^1^H and ^13^C NMR data: see Table 1; HRESIMS: *m/z* 413.1759 [M + H]^+^ (calcd for C_27_H_25_O_4_^+^, 413.1752).

**Table 1:** ^1^H and ^13^C NMR Data of Compound **10** (CDCl_3_; 500 MHz for ^1^H and 126 MHz for ^13^C NMR; δ in ppm)

position^a^	δ_C_, type	δ_H_ (*J* in Hz)^b^
1	129.1, C	
2, 6	127.9, CH	7.36, *d* (8.5)
3, 5	114.9, CH	6.93 *d* (8.5)
4	158.7 C	
7	134.1, CH	6.67 *d* (15.9)
8	121.0, CH	6.25
9	65.4 CH_2_	4.86 dd (6.1, 0.9)
1′	134.4 C	
2′, 6′	128.1 CH	7.54 *m*
3′, 5′	128.9 CH	7.39
4′	130.3 CH	7.39
7′	145.0 CH	7.74 *d* (16.2)
8′	118.0 CH	6.49 *d* (15.9)
9′	166.8 C	
1″	129.1 C	
2″, 6″	128.0 CH	7.31 *d* (8.5)
3″, 5″	1115.4 CH	6.80 *d* (8.5)
4″	155.4 C	
7″	132.9 CH	6.67 *d* (15.9)
8″	121.9 CH	6.28
9″	68.8 CH_2_	4.69 dd (6.1, 1.2)

a For atom numbering, see Figure 3.

b Overlapping signals are reported without multiplicity.

### Quantification of *p*-Coumaryl Cinnamate

Analysis was performed on a chromatographic system consisting of a Waters 2695 Alliance Separation Module equipped with a Waters 996 photodiode array detector. Separations were carried out on a SunFire C_18_ column (3.5 μm, 150 × 3.0 mm i.d.) equipped with a guard column (10 mm × 3.0 mm i.d.) (Waters, Milford, MA, USA). A 15 min-gradient of 55–70% acetonitrile in water, both containing 0.1% formic acid, was applied at a flow rate of 0.45 mL/min. Detection wavelength was set at 280 nm. Empower 2 software was used for data acquisition and processing (all Waters, Milford, MA, USA).

Sumatra benzoin samples (0.500 g ± 0.005 g) were exactly weighted into 15 mL Falcon tubes and dissolved with 96% ethanol to a concentration of 0.05 g/mL. The solution was then centrifuged at 3000 rpm for 10 min. For analysis, 20 μL of the supernatant was diluted with 980 μL of 96% ethanol. As *p*-coumaryl cinnamate is prone to hydrolysis, samples were prepared in batches of 12, and the temperature of the autosampler was set to 4 °C. For the reference grade A resin, three separate samples prepared with the method described above were analyzed. Quantification was performed with the external standard method. A calibration curve was made with *p*-coumaryl cinnamate (*t*_R_ = 8.5 min), isolated in the course of this study, at concentrations ranging from 0.025 to 0.250 mg/mL (quantification in reference grade A Sumatra benzoin) or 0.001 to 0.4 mg/mL (quantification in the large set of resins) in 96% ethanol. Limit of detection (LOD, S/N > 3) and limit of quantification (LOQ, S/N > 10) were determined to be 0.075 and 0.2 μg/mL, respectively. Reproducibility was checked by repeated injection (*n* = 6) of *p*-coumaryl cinnamate [50 and 200 μg/mL, relative standard deviation (RSD) ≤ 1] and injection of resin samples of different grades (*n* = 1) at 2 h intervals for a total of 10 h (RSD ≤2%). A volume of 10 μL was injected for calibrations and test samples.

### Quantification of Sumaresinolic Acid

Sumaresinolic acid was quantified by HPLC-ELSD analysis. The instrumentation and the chromatographic condition were as described above for HPLC-PDA-ELSD-ESIMS analysis. Three independently prepared samples of reference grade A resin (50.0 mg) were dissolved with methanol in volumetric flasks to a concentration of 1 mg/mL. The samples were filtered through a 0.45 μm PTFE syringe filter (Simplepure, USA) before analysis. Quantification was done by the external standard method. A calibration curve was prepared with sumaresinolic acid (*t*_R_ = 37.6 min), isolated in the course of this study, at concentrations ranging from 0.025 to 0.250 mg/mL in MeOH. A volume of 8 μL was injected for all calibrators and test samples.

### Statistical Analysis

Student’s t-test was used to test for statistically significant differences. All statistical analyses were performed using the SPSS statistical package (version 27.0, IBM, Chicago, IL, USA). *P*-values of <0.05 were considered statistically significant.

## Results and Discussion

### Chemical Profile of Sumatra Benzoin Reference Grade A

**Figure 1:**
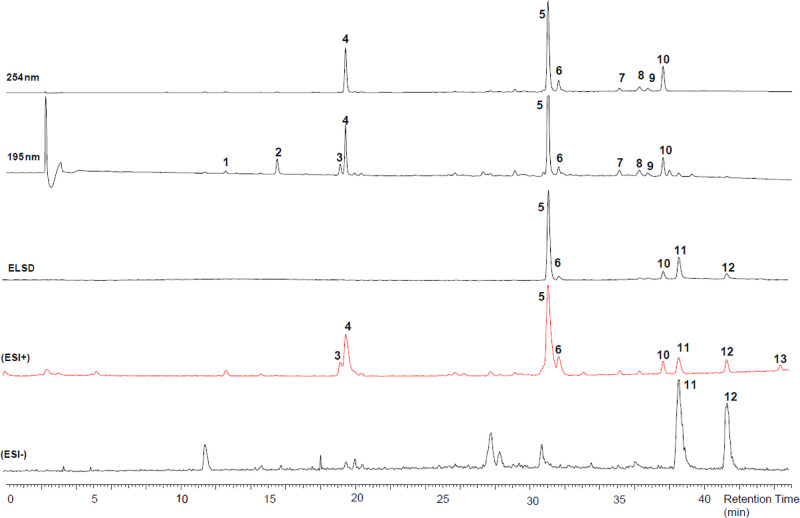
HPLC-PDA-ELSD-ESIMS analysis of Sumatra benzoin grade A. Chromatographic conditions: SunFire C_18_ column; 5–100% MeCN in water (both containing formic acid 0.1%) in 45 min; 0.4 mL/min. Identified compounds are numbered accordingly. ELSD: evaporative light scattering detector, BPC: Base peak chromatogram.

**Figure 2:**
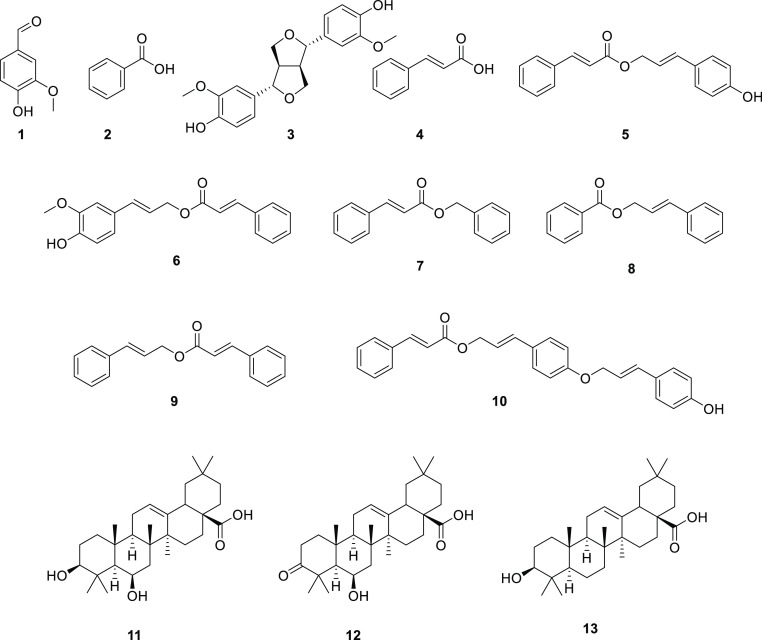
Structures of compounds **1–13**.

The chemical profile of a reference grade A sample of Sumatra benzoin was established by HPLC-PDA-ELSD-ESIMS analysis (Figure 1). A total of 13 compounds (Figure 2) corresponding to the major peaks detected by the different detectors were identified after preparative isolation by ESIMS and NMR analysis (Tables S1–S3). They included compounds which had been previously identified in Sumatra benzoin such as vanillin (**1**),^13^ benzoic acid (**2**),^13^ pinoresinol (**3**),^16^ cinnamic acid (**4**),^13^*p*-coumaryl cinnamate (**5**),^8^ coniferyl cinnamate (**6**),^8^ benzyl cinnamate (**7**),^8^ cinnamyl benzoate (**8**),^8^ cinnamyl cinnamate (**9**),^8^ sumaresinolic acid (**11**),^7,8^ and oleanolic acid (**13**),^7,19^ as well as 6β-hydroxy-3-oxo-12-oleanen-28-oic acid (**12**), previously reported in Siam benzoin.^7^ In addition, a new secondary metabolite **10** was isolated and its structure established*.*

**Figure 3:**
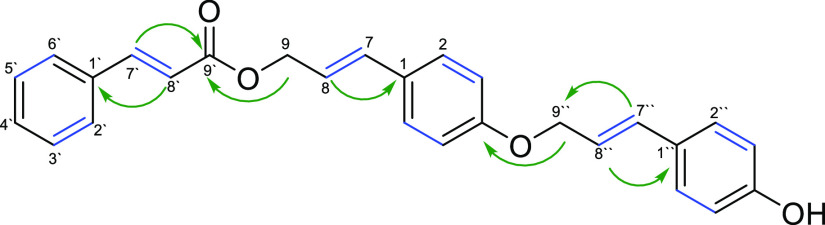
Key HMBC (green arrows) and COSY (blue bonds) correlations for compound **10**.

Compound **10** was obtained as a yellow resin. Its molecular formula was established as C_27_H_24_O_4_ from the quasi-molecular [M + H]^+^ ion in HRESIMS (*m/z* 413.1759; calcd for C_27_H_25_O_4_^+^, 413.1752). ^1^H and ^13^C NMR spectra (Table 1) showed characteristic resonances which indicated the presence of one monosubstituted and two *p*-disubstituted phenyl rings, three pairs of olefinic protons, two oxygenated methylene groups, and an ester carbonyl. A detailed comparison with the NMR spectra of **5** revealed that compound **10** differed from the latter by the presence of an additional *p*-coumaryl moiety. The downfield shift of C-4 (δ_C_ 158.7) in compound **10** (δ_C_ 155.9 in **5**) suggested the attachment of the additional *p*-coumaryl moiety at C-4. A heteronuclear multiple bond correlation (HMBC) between H-9″ and C-4 unambiguously established the attachment of the second *p*-coumaryl moiety at C-4 through an ether bond with its alcoholic group and established the structure as shown in Figure 2. Key HMBC and correlation spectroscopy (COSY) correlations are given in Figure 3.

**Figure 4:**
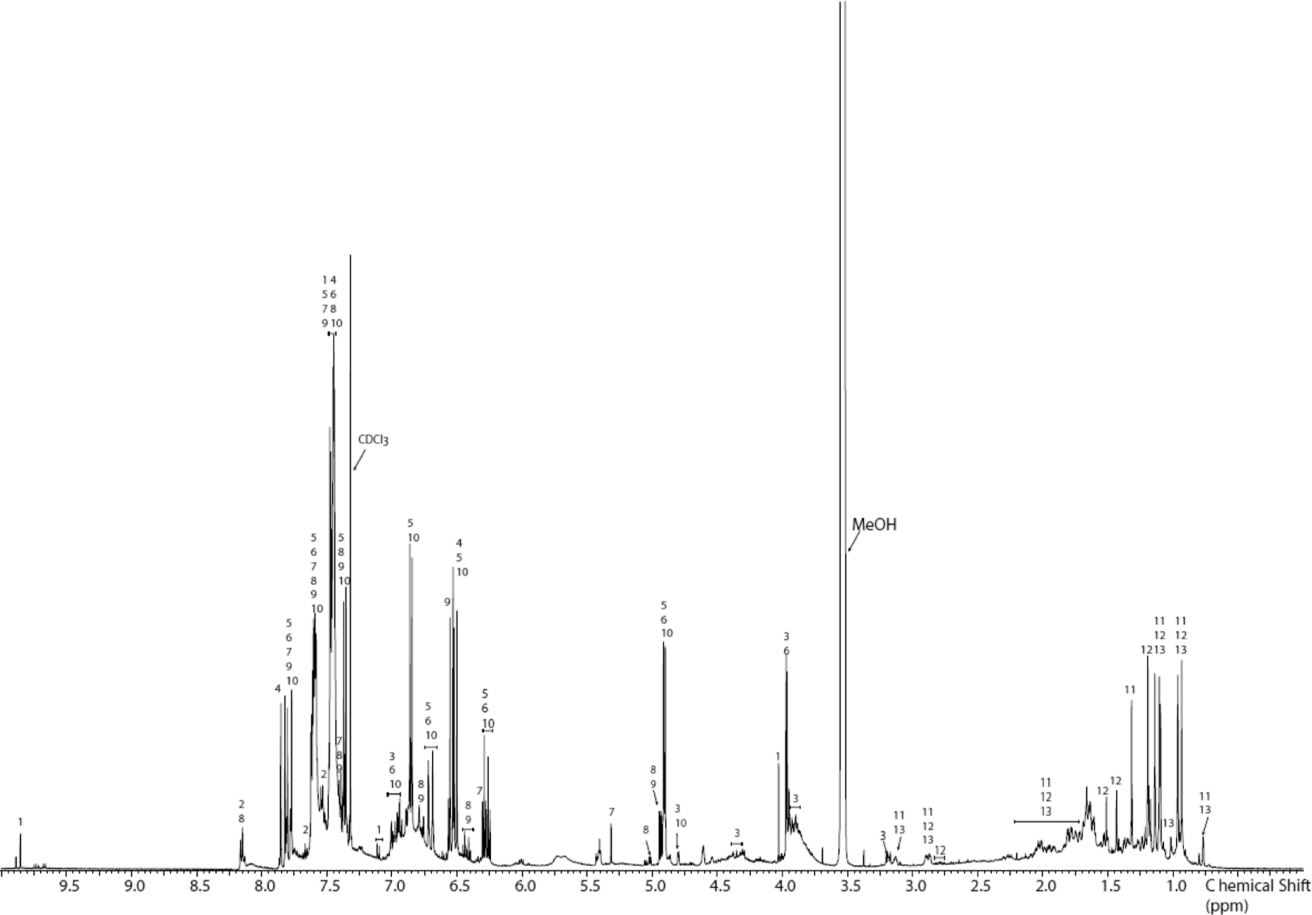
^1^H NMR spectrum of Sumatra benzoin reference grade A (500 MHz, CDCl_3_). Peaks are labeled with numbers corresponding to the isolated compounds.

To confirm that the identified compounds accounted for the major part of the resin composition, we recorded a ^1^H NMR spectrum of Sumatra benzoin in CDCl_3_ and correlated the signals with those observed in the spectra of the purified compounds recorded in the same solvent (Figure 4). The strong signals between 6.25 and 8.0 ppm and around 5 ppm in the ^1^H NMR spectrum could be assigned to the phenolic ester derivatives. A second main group of signals was observed in the aliphatic region and could be attributed to the triterpenoids. A highly distinctive feature was the singlets between 0.9 and 1.5 ppm of the angular methyl groups. Importantly, most of the signals, including all signals of noticeable intensity, could be assigned to the identified compounds, despite overlapping in the aliphatic region of unresolved triterpenoid signals. Based on these data, it could be estimated that compounds **1–13** accounted for approx. 90% of the organic compounds in Sumatra benzoin.

### Quantitative Determination of *p*-Coumaryl Cinnamate and Sumaresinolic Acid in Reference Grade A Sumatra Benzoin

In a next step, we quantified *p*-coumaryl cinnamate (**5**) and sumaresinolic acid (**11**) in Sumatra benzoin grade A reference resin. According to HPLC and NMR analysis, phenolic esters are the largest group of constituents in the resin, with *p*-coumaryl cinnamate (**5**) being the major congener. Compound **5** was quantified in Sumatra benzoin grade A by HPLC-UV analysis with the aid of a calibration curve from 25 to 250 μg/mL (*R*^2^ 0.99). Analysis of three samples of powdered Sumatra benzoin grade A prepared separately afforded a content of 212.9 ± 3.2 mg/g of **5** in the resin.

In the ^1^H NMR spectrum of Sumatra benzoin (Figure 4), the strong signals in the upfield region indicated the presence of significant amounts of triterpenes **11–13**. HPLC-ELSD analysis (Figure 1) indicated sumaresinolic acid (**11**) to be the major congener. The compound was thus quantified by HPLC-ELSD analysis with a calibration curve from 25 to 250 μg/mL (*R*^2^ 0.99). Three samples of powdered Sumatra benzoin grade A were separately prepared and analyzed. A content of 81.8 ± 2.3 mg mg/g of **11** in the resin was determined.

### Quantification of *p*-Coumaryl Cinnamate in a Large Set of Resin Samples

**Figure 5:**
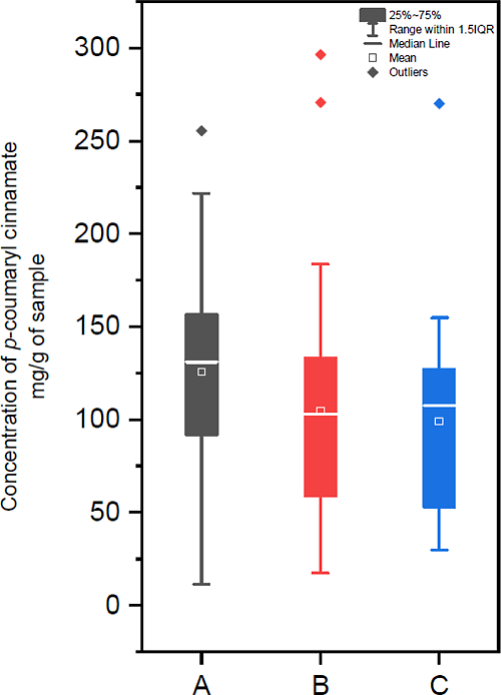
Box-plot diagram of *p*-coumaryl cinnamate (**5**) concentration (mg/g) for 118 samples grouped according to their respective grades A (*n* = 45), B (*n* = 45), and C (*n* = 28).

Due to its antifungal properties, the content of *p*-coumaryl cinnamate (**5**) is pivotal in view of potential use of the resin as an agricultural pesticide. To investigate the variation of the content of **5** in resins of different origins and grades, a total of 118 Sumatra benzoin samples were analyzed. Since the compound is possibly sensitive to hydrolysis, stability was evaluated by repeated analysis of a standard solution of 0.10 mg/mL at 0, 2, 4, 6, 8, and 10 h intervals. Results revealed no degradation of **5** over this time window (SD < 1%). To further exclude possible degradation in the autosampler, resin samples were prepared in batches of 12. System reproducibility tests were performed with the aid of repeated injections of 50 and 200 μg/mL solutions, resulting with a standard deviation value at < 1%. Two calibration curves of **5** recorded in the course of the analysis revealed good linearity, with a *R*^2^ value >0.99. The content of **5** in grade A, B, and C resins was determined to be 127.3 ± 54.1, 104.6 ± 58.8, and 98.9 ± 51.1 mg/g, respectively (Figure 5). Statistical analysis indicated no significant difference between the contents in grade A and grade B or between grade B and grade C samples (*P* > 0.05). However, a significant difference was found when comparing samples from grade A and grade C (*P* < 0.05). The large standard deviation values indicated a large variability of the *p*-coumaryl cinnamate (**5**) content within each grade of resin. Nevertheless, when looking at the interquartile range between the 25 to 75 percentile, grade A resin showed the lowest spread (Figure 5), followed by grade C and grade B. Most grade A samples had a content of **5** between 91.8 and 163.7 mg/g, but there were notable outliers with extremely low or high content, such as samples 41A (255 mg/g) and 21A (11 mg/g).

### Comparison of the Full Chemical Profile of Resin Samples from Different Grades and Origins

**Figure 6:**
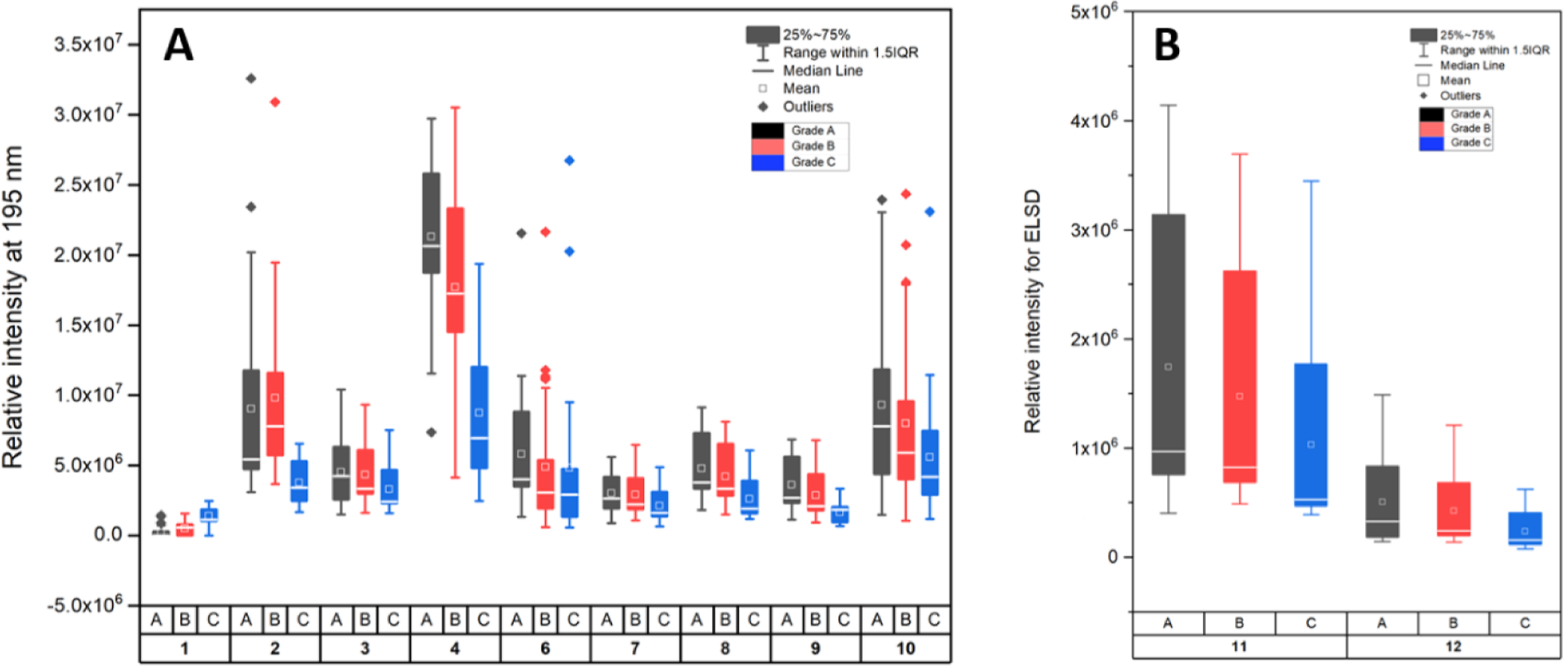
Box-plot of the relative intensity of compounds **1–12** in 81 samples grouped according to their respective grades A (*n* = 27), B (*n* = 27), and C (*n* = 27). A: intensity obtained with UV detection at 195 nm; B: Intensity obtained from ELSD.

To evaluate the variability in the full chemical profiles between resin samples of different origins and grades, we analyzed 81 samples by HPLC-PDA-ELSD-ESIMS. The method described for the chemical profiling of the reference grade A resin was used. The samples were selected from groups with complete sets of A, B, and C graded resin (see Supporting Information). A relative quantitative determination of eleven compounds (**1**–**4** and **6**–**12**) was performed utilizing either UV detection at 195 nm and/or ELSD (Figure 6). The mean concentration of compounds **3**, **4**, and **6**–**11** followed a trend of having the highest mean concentration in grade A samples, followed by grade B and finally grade C. However, this trend was not observed for compounds **1** and **2**. Statistical analysis showed that there were no statistically significant differences in the relative intensities of all compounds between grade A and grade B samples (*P* > 0.05). However, seven (**2**, **3**, **8**–**11**, and **12**) out of the eleven compounds showed a significant difference between grade A and grade C. Finally, a significant difference was observed for six compounds (**1**, **2**, **8**, **9**, **11**, and **12**) between grade B and grade C. Full statistical data are shown in Figure S4.

**Figure 7:**
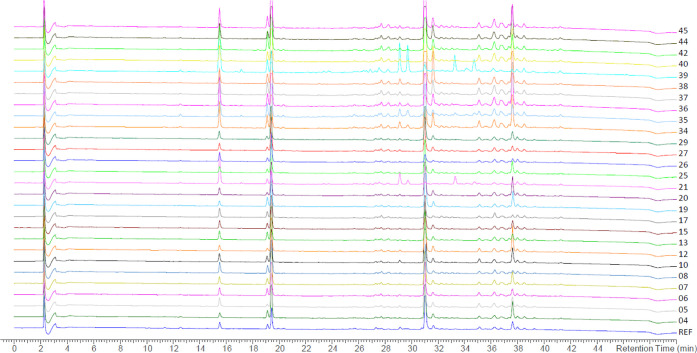
HPLC-PDA analysis of grade A resin samples. Chromatographic conditions: SunFire C_18_; 5–100% MeCN in water (both containing formic acid 0.1%) in 45 min; 0.4 mL/min, detection at 195 nm.

To investigate the variation between samples of different origins, we compared the subset of grade A samples. An overlay of the chromatographic traces recorded at 195 nm of all grade A samples (*n* = 27) in comparison to the grade A reference sample is provided in Figure 7. Almost all samples showed a very similar qualitative chromatographic profile with the different detection methods used. However, a few samples exhibited some additional peaks. Compared to reference resin and other grade A resins, three strikingly stronger signals were detected in sample **21** at *t*_R_ = 29.1 min, *t*_R_ = 29.7 min, and *t*_R_ = 33.2 min. Sample **39** also displayed four salient peaks at *t*_R_ = 29.1 min, *t*_R_ = 29.7 min, *t*_R_ = 33.2 min, and *t*_R_ = 34.7 min. The UV and mass spectra of the unknown peaks with the same retention times in both samples were similar to each other. Due to the limited amount of the corresponding resin samples, the compounds could not be isolated for full structural characterization. However, the MS patterns and UV spectra of compounds with *t*_R_ = 29.7 min and *t*_R_ = 34.7 min suggest that they may correspond to dimethoxycinnamyl cinnamate isomers, while the peak at *t*_R_ = 33.2 min could be a further unidentified cinnamyl ester congener.

Despite the similar chromatographic profile of most grade A resin samples, considerable differences were observed in the relative content of the constituents. Thus, RSD values of 26% (**4**), 43% (**7**), 45% (**8**), 50% (**3** and **9**) and 74–78% (**2**, **6**, **10**, **11**, and **12**) indicated large variation in the content of these compounds. Interestingly, most resins showed a similar qualitative profile (Figure 7).

In summary, the combination of HPLC-PDA-ELSD-MS analysis and ^1^H NMR spectroscopy established for the first time a comprehensive picture of the composition of a large set of Sumatra benzoin gums. The identified compounds **1-13** accounted for an estimated 90% of the resin. Analysis of a large set of samples from North Sumatra purchased in Indonesia revealed significant differences in the quantitative composition of the resins despite similar qualitative profiles. Differences were observed between quality grades, but other factors, such as harvesting protocols or environmental conditions, may contribute to sample variation. This underlines the need of a thorough quality assessment of Sumatra benzoin for specific uses.

## Supporting Information Available

Isolation scheme of compounds; NMR spectra of compound **10**; NMR data of compounds **1**–**9** and **11**–**13**; comparison of relative intensity of compounds detected in different grades of resin; and HPLC analysis of grade B and grade C samples (PDF)
