# Supplementary material for: Qualitative and Quantitative Secondary Metabolite Profiles in a Large Set of Sumatra Benzoin Samples
Source: J Agric Food Chem. 2023 Jul 3;71(28):10590–7. doi: 10.1021/acs.jafc.3c01861 (PMC10360151; doi:10.1021/acs.jafc.3c01861)
Supplement: Supplementary file 1 — jf3c01861_si_001.pdf [file jf3c01861_si_001.pdf]

Supporting information

Qualitative and Quantitative Secondary Metabolite Profile in a Large Set of Sumatra Benzoin Samples

Ming Yuan Heng[1], Nova Syafni[1,2], Justine Ramseyer[1], Barbara Thuerig[3], Lucius Tamm[3], Matthias Hamburger[1], Olivier Potterat[1*]

[1] Pharmaceutical Biology, University of Basel, CH-4056 Basel, Switzerland

[2] Faculty of Pharmacy and Sumatran Biota Laboratory, Andalas University, Kampus Limau Manis, Padang, West Sumatra 25163, Indonesia

[3] Research Institute of Organic Agriculture FiBL, Ackerstrasse 113, CH-5070 Frick, Switzerland

[*]Corresponding Author

Olivier Potterat – Pharmaceutical Biology, University of Basel, CH-4056 Basel, Switzerland; Phone: +41-61-207-1534; Email: olivier.potterat@unibas.ch; Orcid.org/0000-0001-5962-6516

Contents

Figure S1. Isolation scheme of compounds 3

Figure S2. Structures of compounds **1**-**9** and **11**-**23** with atom numbering 4

Table S1. NMR tables of compound **1** – **5** 5

Table S2. NMR tables of compound **6** – **10** 6

Table S3. NMR tables of compound **11** – **13** 7

Table S4. Comparison of relative intensity of compounds detected in different grades of resin 8

Figure S3. 1H-NMR spectrum of compound **10** (500 MHz, $CDCl_3$) 9

Figure S4. 13C-DEPTq spectrum of compound **10** (126 MHz, $CDCl_3$) 9

Figure S5. 1H-1H COSY spectrum of compound **10** (500 MHz, $CDCl_3$) 10

Figure S6. 1H-1H ROESY spectrum of compound **10** (500 MHz, $CDCl_3$) 10

Figure S7. HSQC-DEPT spectrum of compound **10** (500 MHz, $CDCl_3$) 11

Figure S8. HMBC spectrum of compound **10** (500 MHz, $CDCl_3$) 11

Figure S9. HPLC-PDA analysis at 254 nm of grade A samples 12

Figure S10. HPLC-ELSD analysis of grade A samples 13

Figure S11. HPLC-PDA analysis at 195 nm of grade B samples 14

Figure S12. HPLC-PDA analysis 254 nm of grade B samples 15

Figure S13. HPLC-ELSD analysis of grade B samples 16

Figure S14. HPLC-PDA analysis at 195 nm of grade C samples 17

Figure S15. HPLC-PDA analysis at 254 nm of grade C samples 18

Figure S16. HPLC-ELSD analysis of grade C samples 19

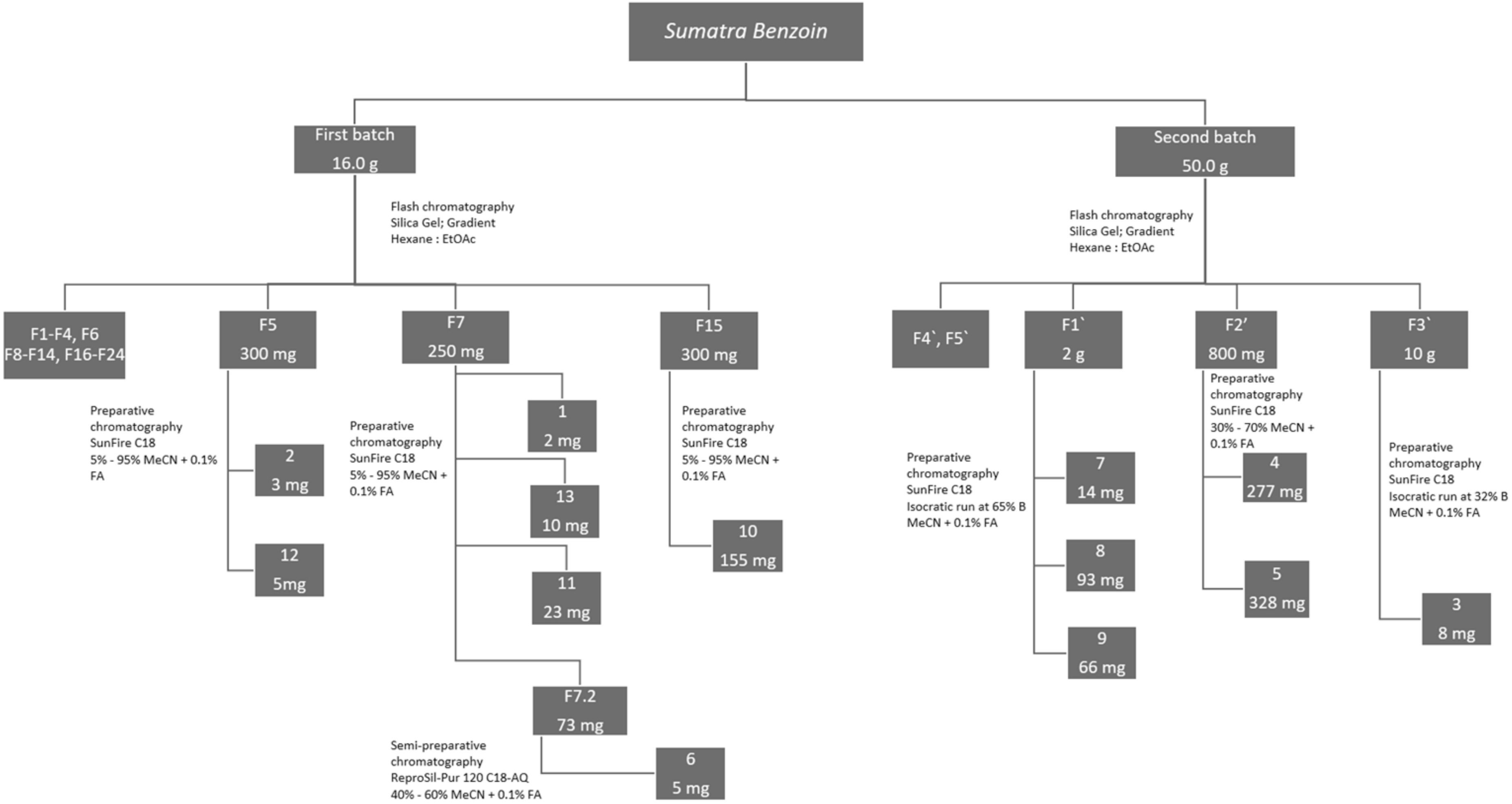

Figure S1. Isolation scheme of compounds

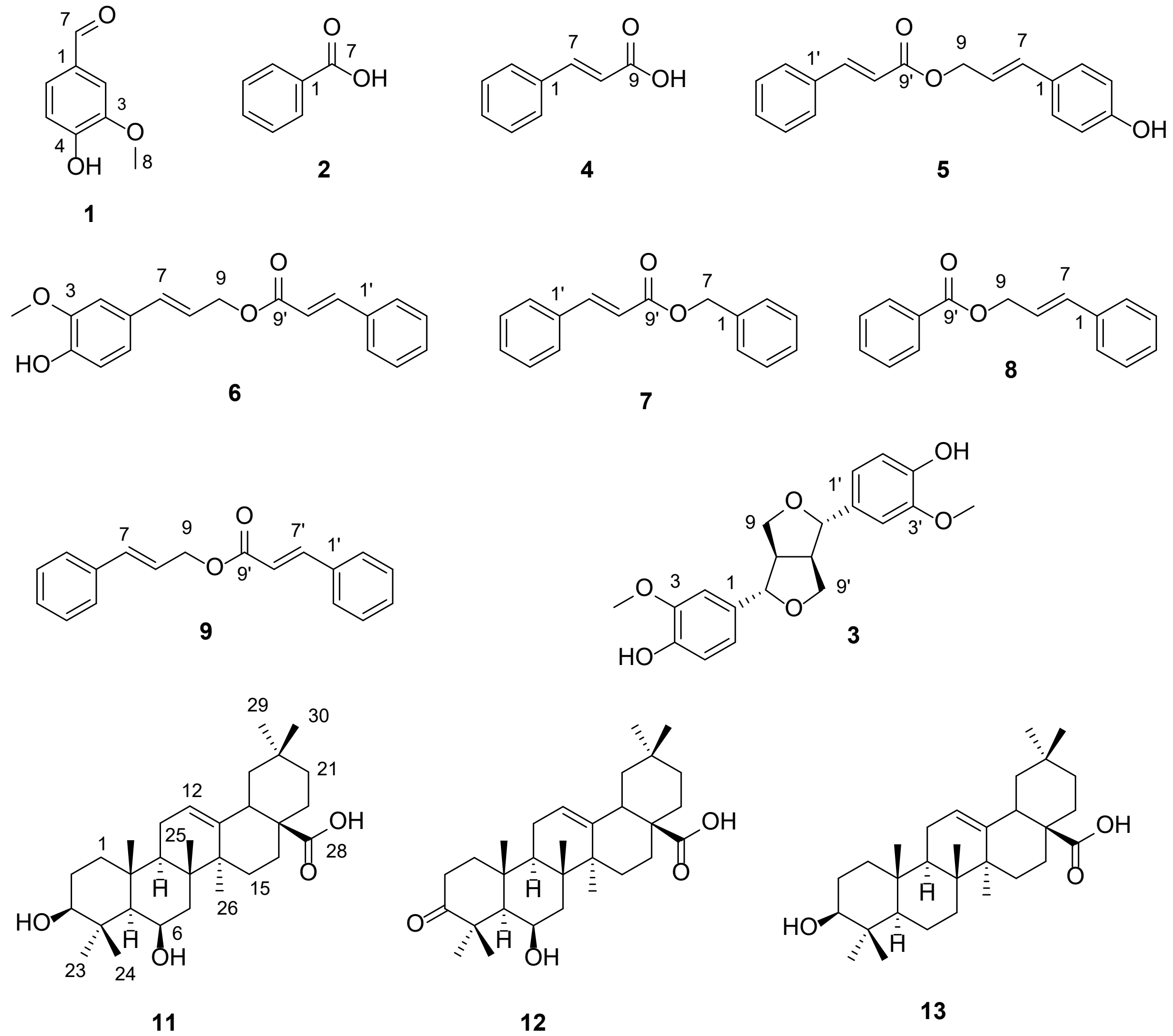

Figure S2. Structures of compounds **1**-**9** and **11**-**13** with atom numbering

Table S1. NMR data of compounds **1** – **5** (500 MHz for ^{1}H and 126 MHz for ^{13}C NMR; δ in ppm)*.

**Position**	Vanillin (**1**) [a]		**Position**	Benzoic acid (**2**) [b]		**Position**	Pinoresinol (**3**)[c]		**Position**	Cinnamic acid (**4**) [a]		**Position**	*p*-Coumaryl cinnamate (**5**)[c]	
	δ_C	δ_H (*J* in Hz)		δ_C	δ_H (*J* in Hz)		δ_C	δ_H (*J* in Hz)		δ_C	δ_H (*J* in Hz)		δ_C	δ_H (*J* in Hz)
1	130.6		1	131.2		1, 1’	132.9		1	135.9		1	129.0	
2	110.9	7.46 br *s*	2,6	129.7	7.95 *m*	2, 2’	108.6	6.91	2, 6	129.3	7.57	2, 6	128.2	7.29 *d* (8.5)
3	149.4		3,5	129.0	7.50 *m*	3, 3’	146.7		3, 5	131.5	7.39	3, 5	115.6	6.82 *m*
4	154.7		4	133.3	7.67 *m*	4, 4’	145.2		4	130.1	7.39	4	155.9	
5	116.1	6.96 *d* (7.9)	7	167.8		5, 5’	114.3	6.90	7	146.5	7.67 *d* (15.9)	7	134.2	6.65 *d* (15.6)
6	127.7	7.45 *dd* (7.9, 1.6)				6, 6’	119	6.83 *dd* (9.2, 1.8)	8	119.4	6.48 *d* (16.2)	8	120.8	6.22 pseudo *dt* (15.9, 6.7)
7	192.5	9.76 *s*				7, 7’	85.9	4.75 *d* (4.3)	9	170.5		9	65.6	4.87 *dd* (5.5, 0.5)
3-OCH_3	56.0	3.94 *s*				8, 8’	54.2	3.11 *m*				10		5.58
						9, 9’	71.7	3.88 *dd* (9.2, 3.7), 4.26 *m*				1`	134.3	
						3-OCH_3	56.0	3.92 *s*				2’, 6’	128.2	7.54 *m*
						3’-OCH_3	56.0	3.92 *s*				3’, 5’	128.9	7.39
												4’	130.4	7.40
												7’	145.3	7.75 *d* (15.9)
												8’	117.9	6.50 *d* (16.2)
												9’	167.2	

*Overlapped signals are reported without multiplicity

[a] CD_3OD, [b] DMSO-d_6, [c] $CDCl_3$

Table S2. NMR data of compounds **6** – **9** ($CDCl_3$; 500 MHz for ^1H and 126 MHz for ^{13}C NMR; δ in ppm)*

**Position**	Coniferyl cinnamate (**6**)		**Position**	Benzyl cinnamate (**7**)		**Position**	Cinnamyl benzoate (**8**)#		**Position**	Cinnamyl cinnamate (**9**)#	
	δ_C	δ_H (*J* in Hz)		δ_C	δH (J in Hz)		δ_C	δ_H (*J* in Hz)		δ_C	δ_H (*J* in Hz)
1	128.9		1	136.0		1	136.2		1	136.3	
2	108.4	6.96	2, 6	128.3	7.44	2, 6	126.7	7.34 *m*	2, 6	128.1	7.55
3	146.6		3, 5	128.9	7.40	3, 5	128.6	7.27 *m*	3, 5	130.4	7.43
4	145.9		4	128.3	7.36	4	128.1	7.20 *m*	4	128.9	7.41
5	114.4	6.89	7	66.4	5.27 *s*	7	134.3	6.67 *d* (15.9)	7	134.3	6.73 *d* (15.9)
6	120.7	6.93	1’	134.4		8	123.3	6.34 *pseudo dt* (15.9, 6.4)	8	123.3	6.39 *pseudo dt* (15.9, 6.4)
7	134.5	6.64 *d* (15.6)	2’, 6’	128.1	7.54	9	65.6	4.91 *d* (6.4, 1.5)	9	65.1	4.90 *dd* (6.6, 1.4)
8	120.9	6.23 *dd* (15.9, 6.7)	3’, 5’	130.4	7.40	1’	130.2		1’	134.4	
9	65.4	4.86 *dd* (7.3, 1.0)	4’	128.3	7.36	2’, 6’	129.7	8.01	2’, 6’	126.7	7.43
3-OCH_3	55.9	3.92 *s*	7’	145.2	7.76 *d* (16.2)	3’, 5’	128.4	7.37	3’, 5’	128.6	7.35
1’	134.5		8’	117.9	6.51 *d* (15.9)	4’	133.0	7.49	4’	128.1	7.29
2’, 6’	128.1	7.54 *d* (3.7)	9’	166.9		7’	166.4		7’	145.1	7.74 *d* (15.9)
3’, 5’	130.4	7.40							8’	117.9	6.52 *d* (15.9)
4’	128.9	7.40							9’	166.8	
7’	145.1	7.74 *d* (15.9)									
8’	118.0	6.49 *d* (15.9)									

*Overlapped signals are reported without multiplicity

Solvent signal overlapped in ^1H NMR. Tentative ^1H NMR referencing.

Table S3. NMR data of compound **11** – **13** (500 MHz for ^{1}H and 126 MHz for ^{13}C NMR; δ in ppm)*

**Position**	Sumaresinolic acid (**11**)[a]		**Position**	6-Hydroxy-3-oxo-12-oleanen-28-oic acid (**12**)[b]		**Position**	Oleanolic acid (**13**)[c]	
	δ_C	δ_H (*J* in Hz)		δ_C	δ_H (*J* in Hz)		δ_C	δ_H (*J* in Hz)
1	41.9	1.67, 1.53	1	41.5	1.33, 1.90	1	38.5	0.90, 1.49
2	29.1	1.08, 1.84	2	34.4	2.28, 2.76	2	27.4	1.45
3	80.2	3.08 *dd* (8.5, 4.2)	3	216.6		3	77.3	3.0 *dd* (9.2, 4.3)
4	37.8		4	48.7		4	37.1	
5	57.3	0.73	5	56.6	1.23	5	55.3	0.68
6	68.9	4.49 *m*	6	69.3	4.48	6	18.5	1.48, 1.31
7	42.4	1.59, 0.97	7	40.2	1.53, 1.71	7	32.9	1.23, 1.41
8	40.8		8	38.5		8	39.3	
9	49.7	1.62	9	47.3	1.68	9	47.6	1.5
10	39.8		10	36.4		10	38.8	
11	24.8	1.92, 2.02	11	23.5	1.98, 2.12	11	23.4	1.8
12	124.1	5.28 *m*	12	122.6	5.35 *m*	12	122.0	5.16 *m*
13	144.6		13	143.0		13	144.3	
14	43.5		14	42.4		14	41.8	
15	28.3	1.71, 1.56	15	27.6	1.65, 1.98	15	27.7	0.97, 1.65
16	24.4	1.99, 1.60	16	23.0	1.10, 1.73	16	23.1	1.48, 1.90
17	47.8		17	46.5		17	46.0	
18	43.1	2.87 *d* (14.3)	18	41.0	2.86 *d* (14.9)	18	41.3	2.74 *d* (14.7)
19	47.6	1.71, 1.14	19	45.8	1.18, 1.64	19	46.2	1.04, 1.61
20	31.8		20	30.7		20	30.9	
21	33.7	1.39 *td* (13.8, 4.1), 1.21	21	33.8	1.36, 1.24	21	33.8	1.15, 1.32
22	34.1	1.74, 1.54	22	32.2	1.77, 1.61	22	32.6	1.44, 1.61
23	28.7	1.04[e] *s*	23	25.9	1.17[e] *s*	23	28.7[e]	0.89[e] *s*
24	17.6	1.30[e] *s*	24	23.9	1.42 *s*	24	16.5	0.67 *s*
25	19.1	1.09 *s*	25	16.5	1.49 *s*	25	15.6	0.85 *s*
26	17.9	1.16 *s*	26	18.6	1.16 *s*	26	17.3	0.72 *s*
27	26.7	1.13 *s*	27	25.9	1.11 *s*	27	26.1	1.09 *s*
28	182.0		28	183.8		28	179.1	
29	33.9	0.91 *s*	29	33.1	0.91 *s*	29	33.3	0.87 *s*
30	24.3	0.95 *s*	30	24.8	0.94 *s*	30	23.8	0.87 *s*

*Overlapped signals are reported without multiplicity

[a] CD_3OD, [b] $CDCl_3$, [c] DMSO-d_6

Table S4. Comparison of relative intensities of compounds **1-12** detected in different grades of resin. (+) indicates a significant difference between the values ($P > 0.05$).

	Comparison between grades		
	A:B	A:C	B:C
Vanillin (**1**)			+
Benzoic acid (**2**)		+	+
Pinoresinol (**3**)		+	
Cinnamic acid (**4**)			
p-Coumaryl cinnamate (**5**)		+	
Coniferyl cinnamate (**6**)			
Benzyl cinnamate (**7**)			
Cinnamyl benzoate (**8**)		+	+
Cinnamyl cinnamate (**9**)		+	+
Cinnamate di-coumaryl ether (**10**)		+	
Sumaresinolic acid (**11**)		+	+
6-Hydroxy-3-oxo-12-oleanen-28-oic acid (**12**)		+	+

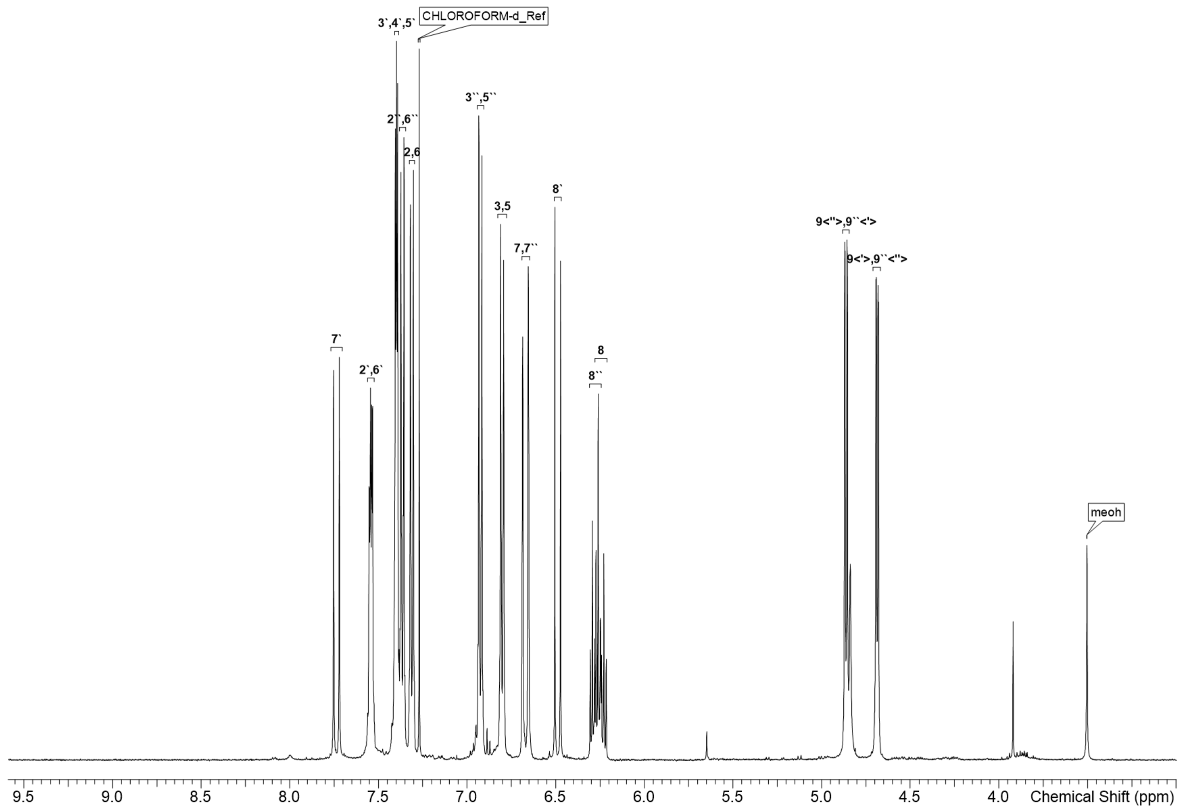

Figure S3. ^{1}H-NMR spectrum of compound **10** (500 MHz, $CDCl_3$).

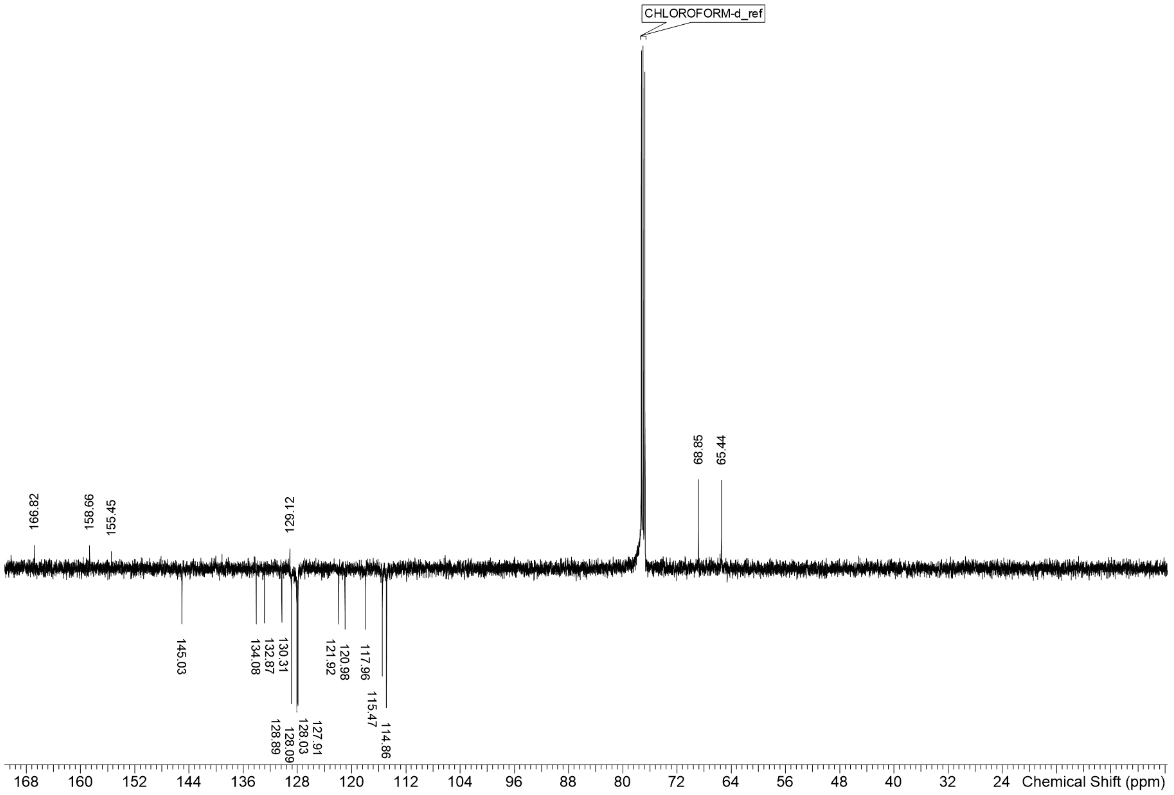

Figure S4. ^{13}C-DEPTq spectrum of compound **10** (126 MHz, $CDCl_3$).

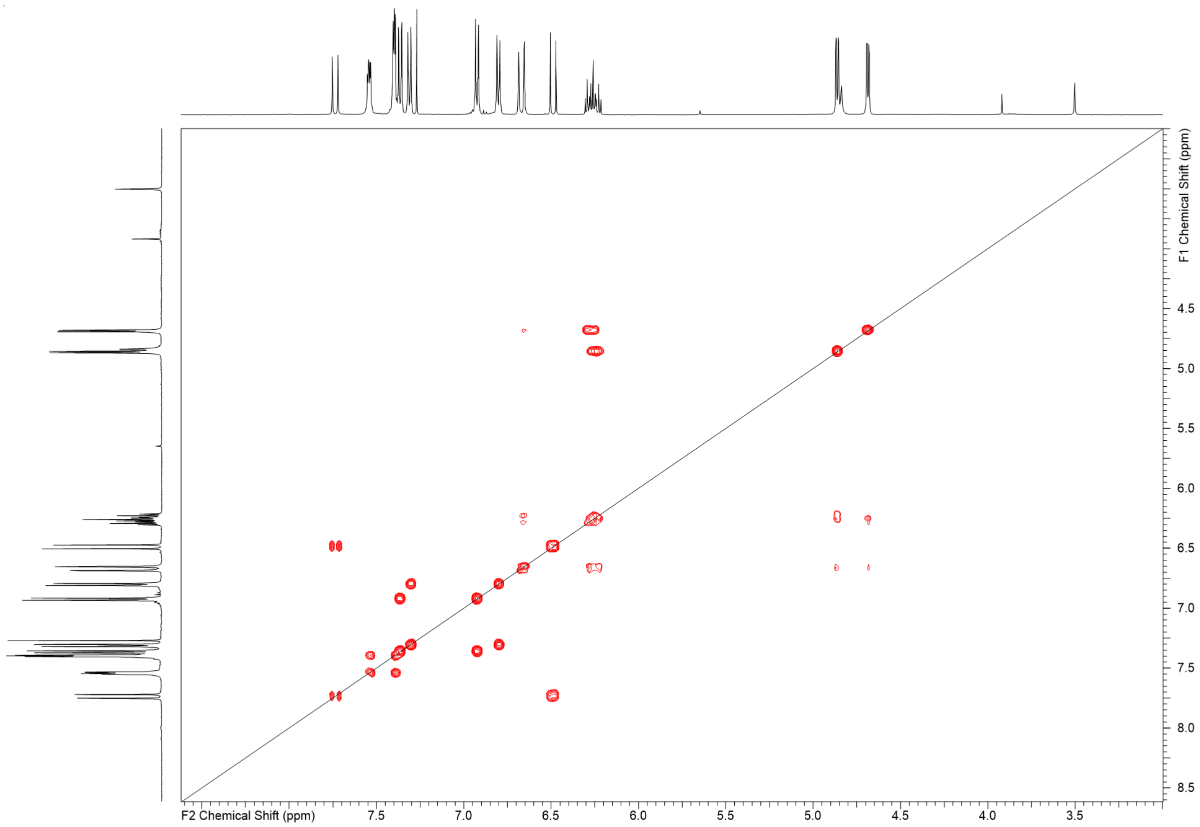

Figure S5. ^1H-^1H COSY spectrum of compound **10** (500 MHz, $CDCl_3$).

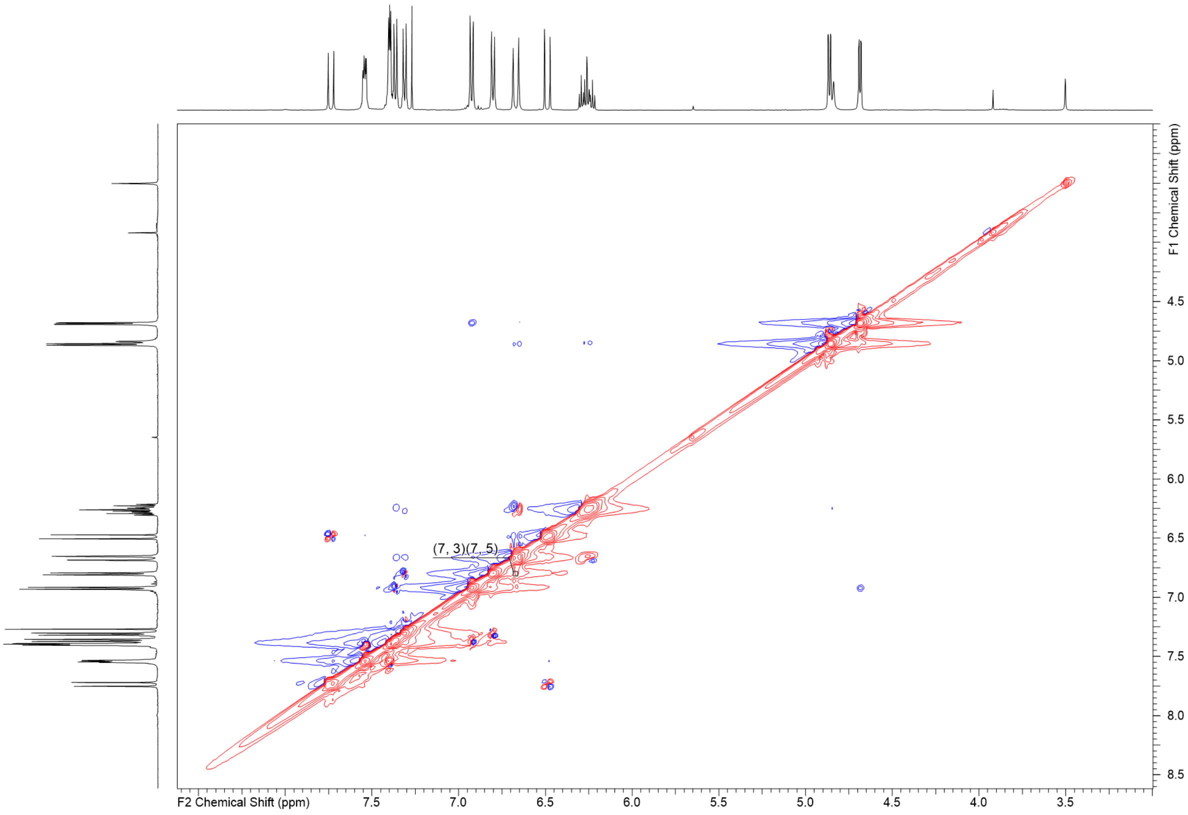

Figure S6. ^1H-^1H ROESY spectrum of compound **10** (500 MHz, $CDCl_3$).

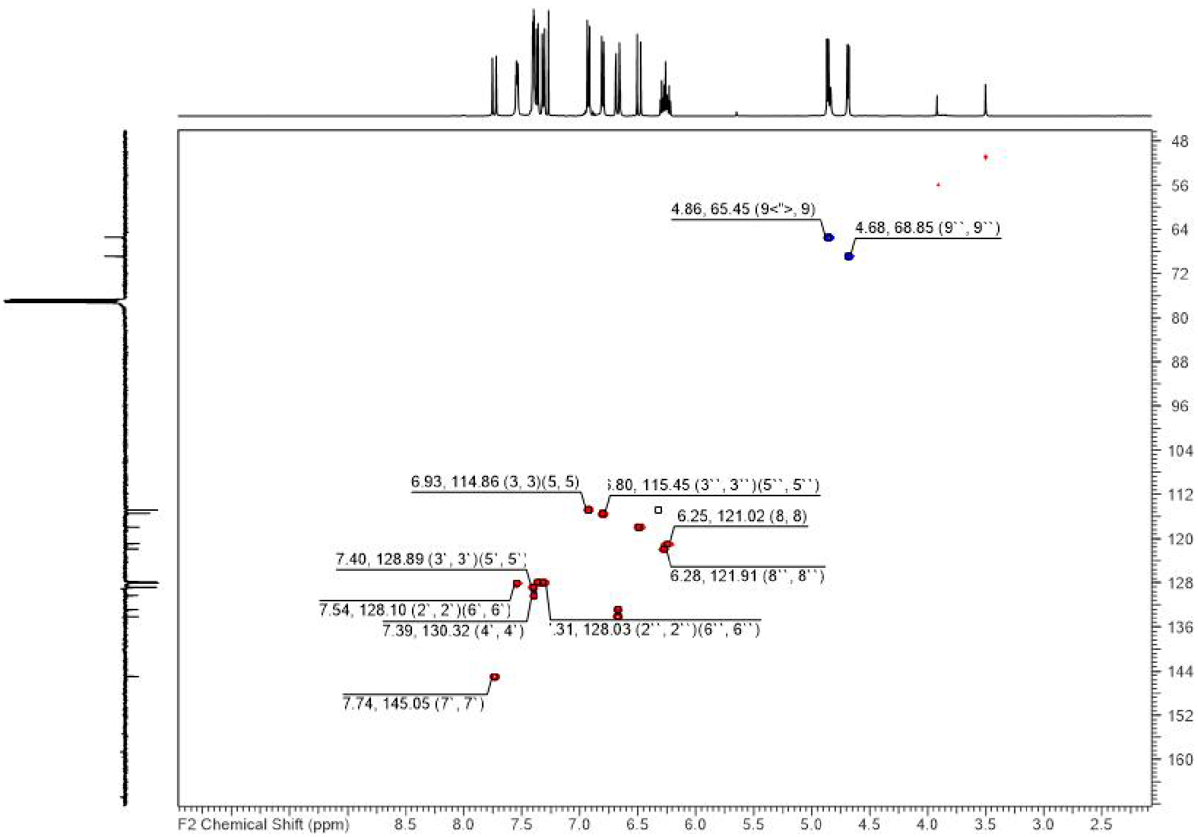

Figure S7. HSQC-DEPT spectrum of compound **10** (500 MHz, $CDCl_3$).

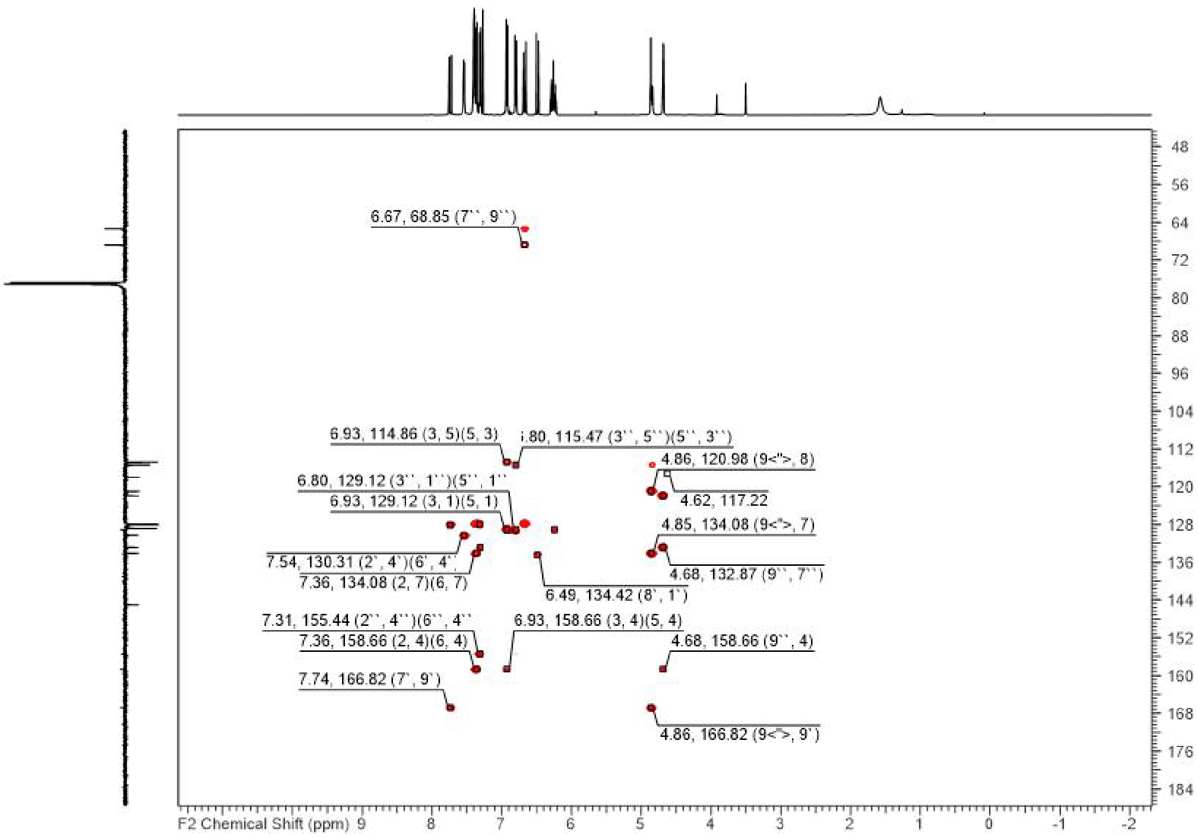

Figure S8. HMBC spectrum of compound **10** (500 MHz, $CDCl_3$).

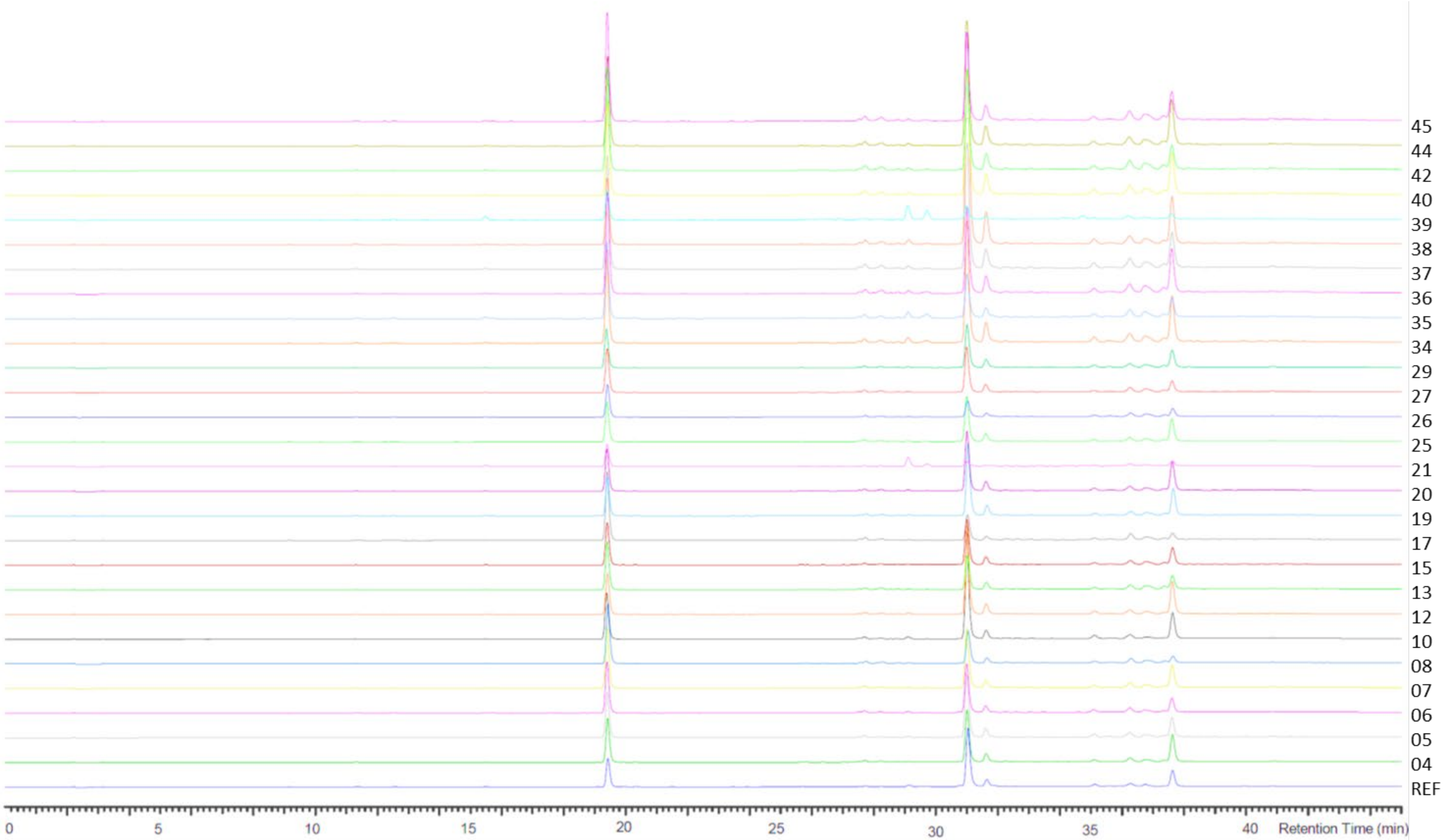

Figure S9. HPLC-PDA analysis at 254 nm of grade A samples. Chromatographic conditions: SunFire C18; 5%-100% MeCN in water (both containing formic acid 0.1%) in 45 min; 0.4 mL/min.

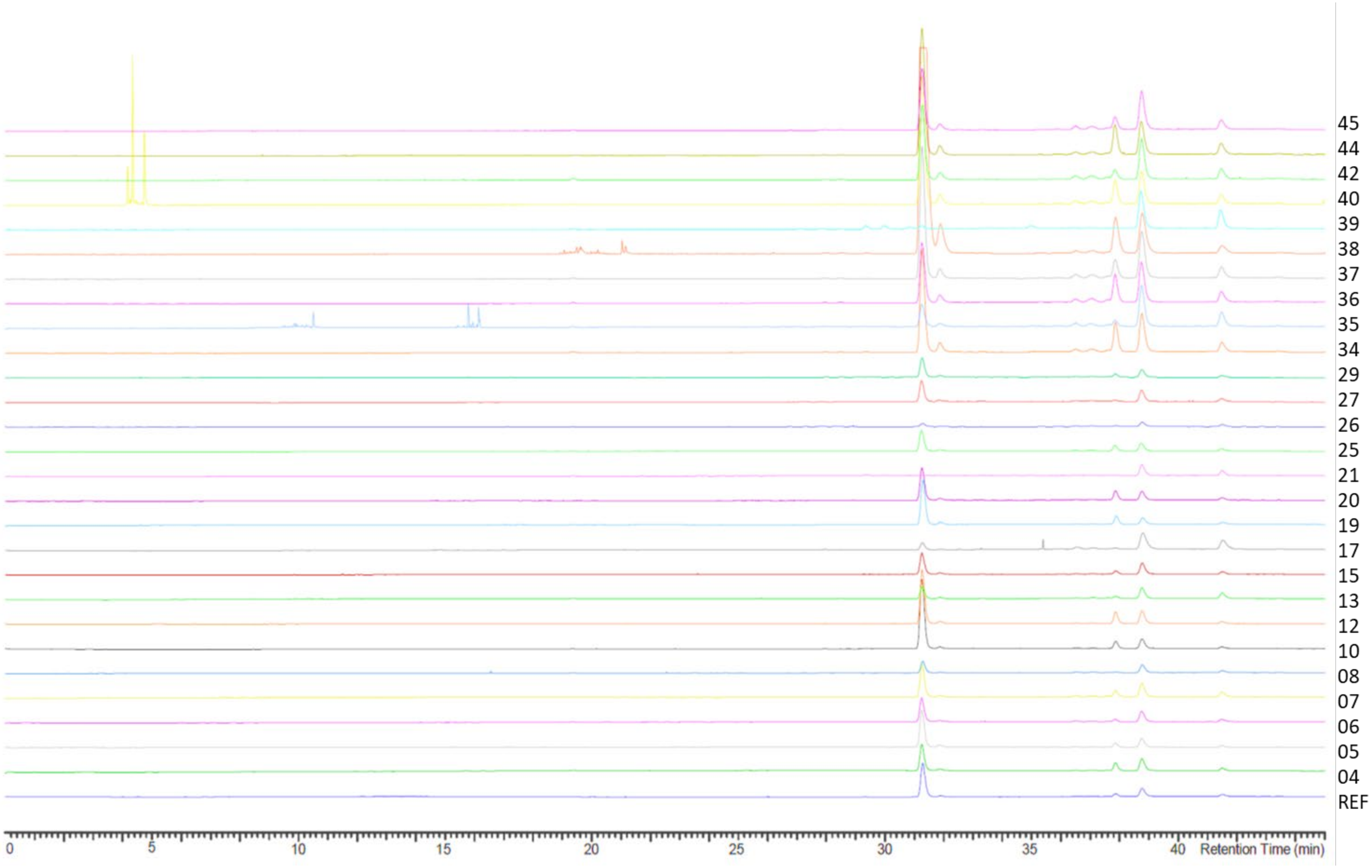

Figure S10. HPLC-ELSD analysis of grade A samples. Chromatographic conditions: SunFire C18; 5%-100% MeCN in water (both containing formic acid 0.1%) in 45 min; 0.4 mL/min

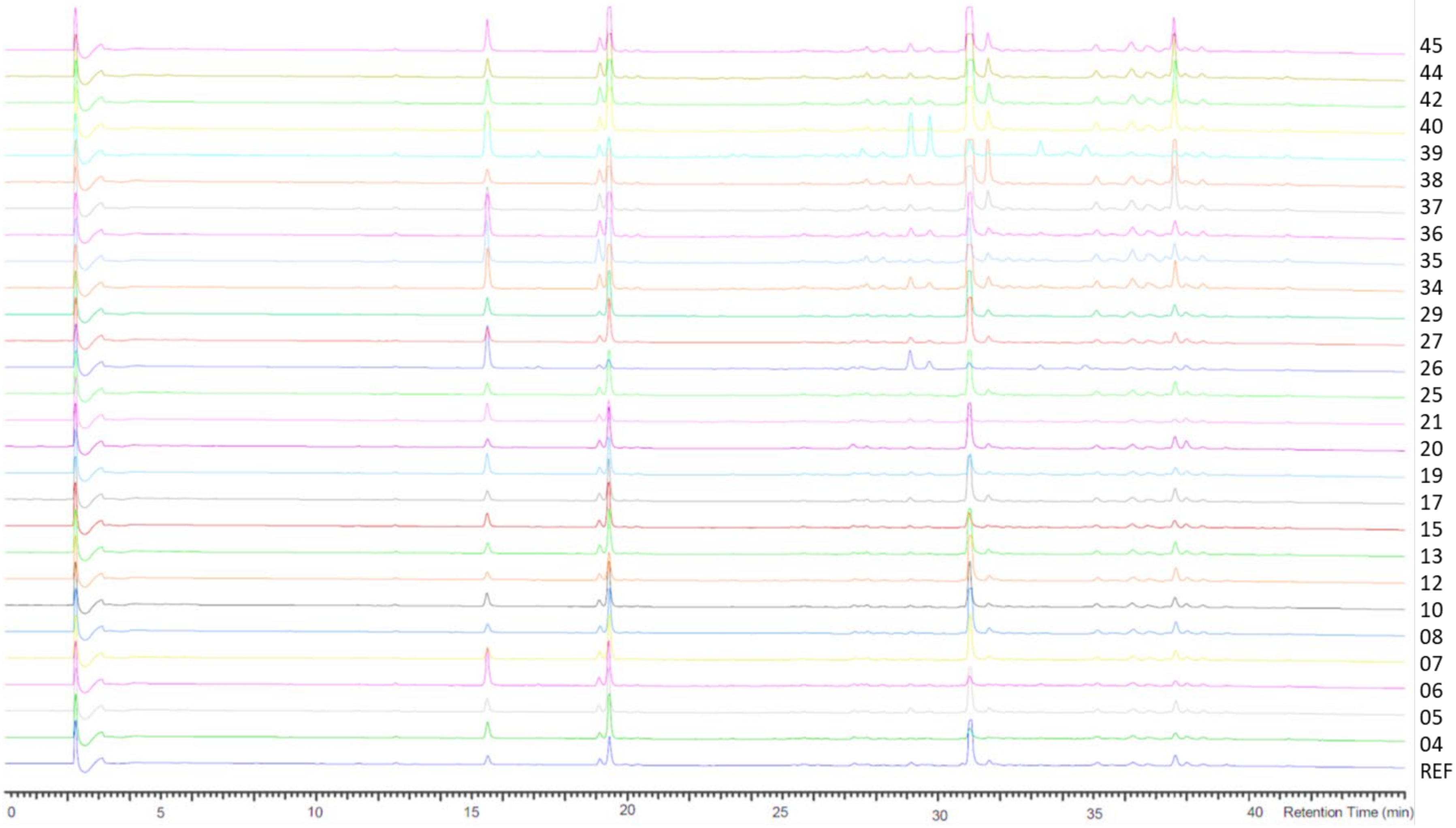

Figure S11. HPLC-PDA analysis at 195 nm of grade B samples. Chromatographic conditions: SunFire C18; 5%-100% MeCN in water (both containing formic acid 0.1%) in 45 min; 0.4 mL/min.

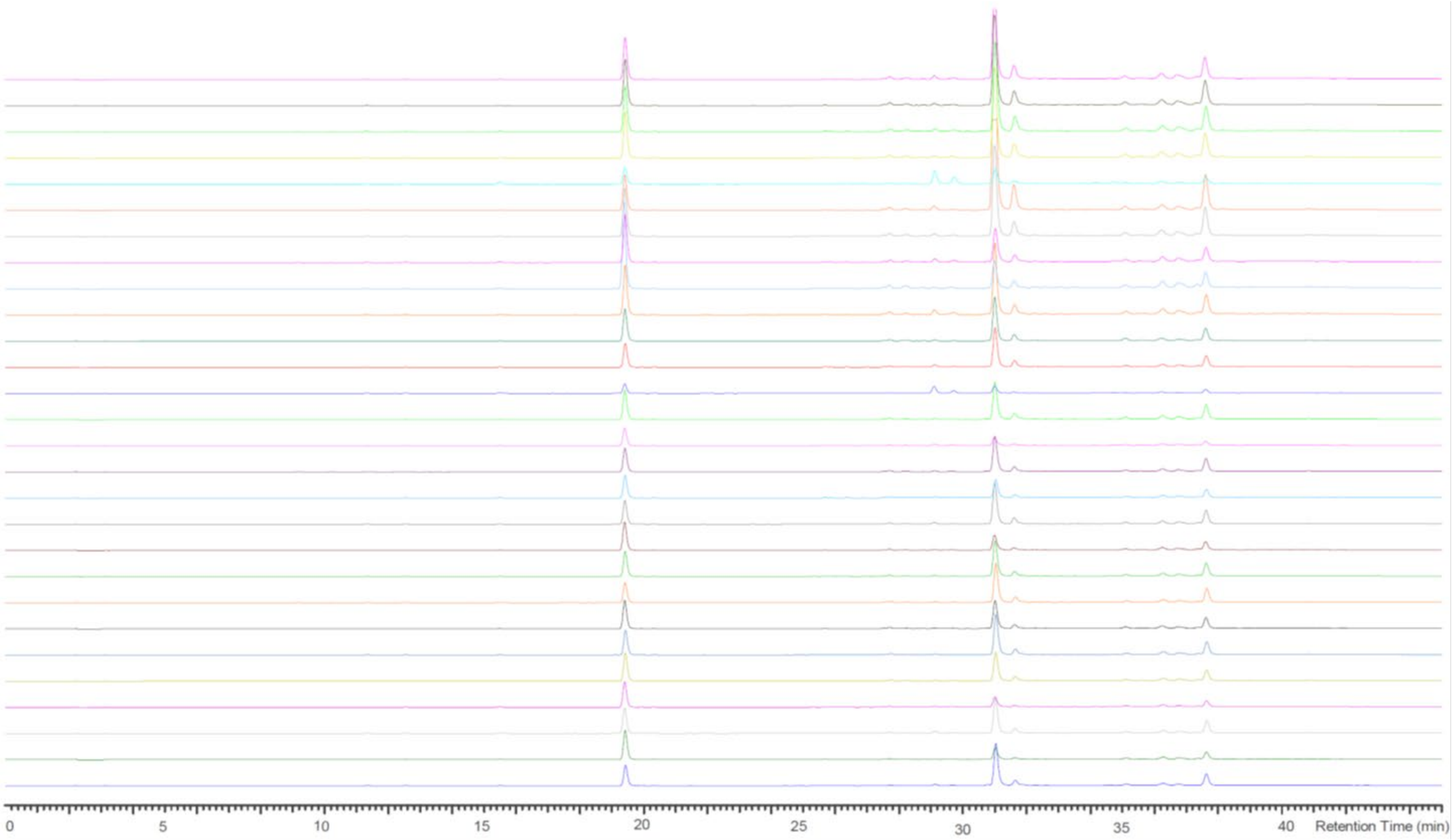

Figure S12. HPLC-PDA analysis at 254 nm of grade B samples. Chromatographic conditions: SunFire C18; 5%-100% MeCN in water (both containing formic acid 0.1%) in 45 min; 0.4 mL/min.

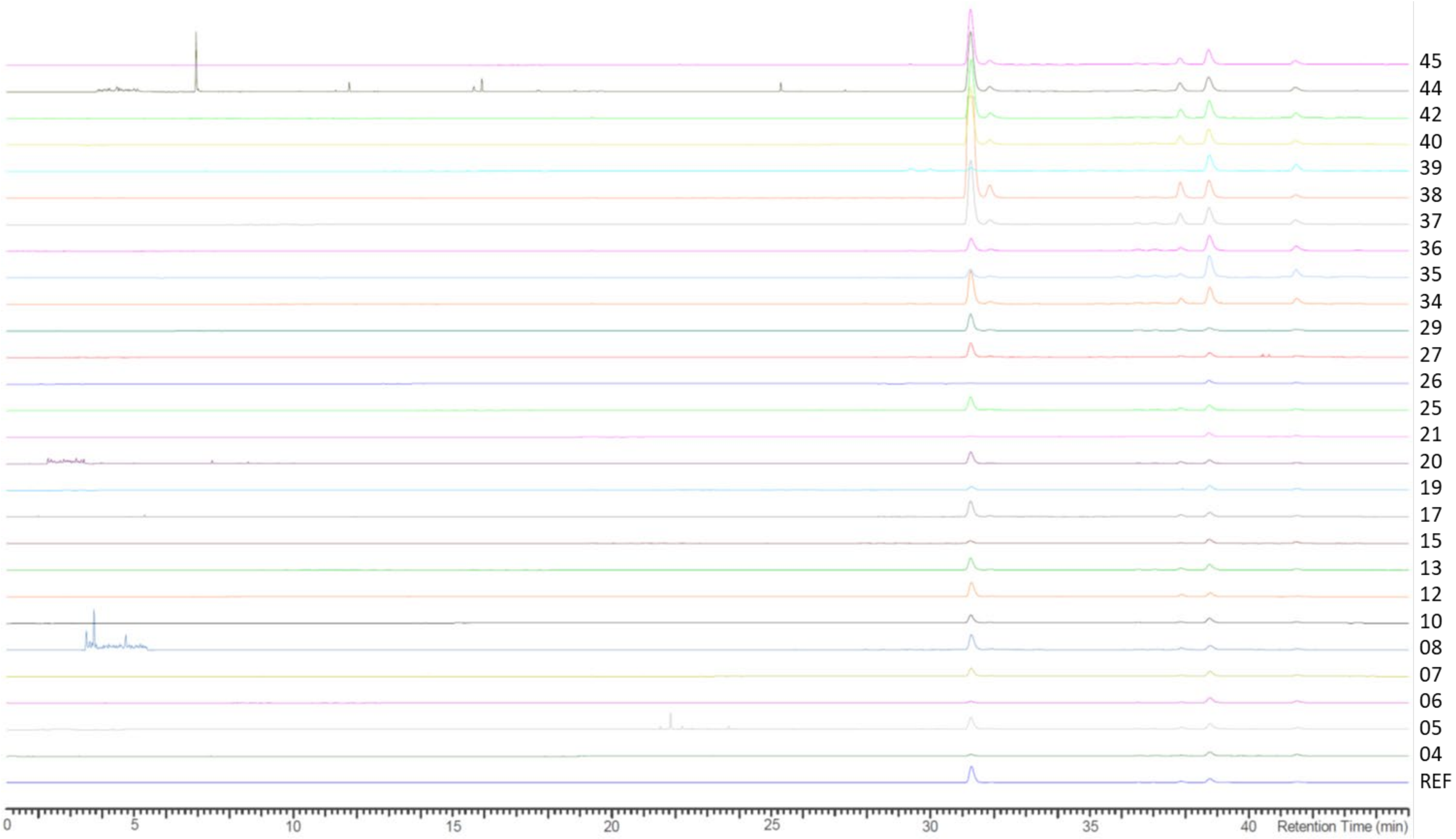

Figure S13. HPLC-ELSD analysis of grade B samples. Chromatographic conditions: SunFire C18; 5%-100% MeCN in water (both containing formic acid 0.1%) in 45 min; 0.4 mL/min.

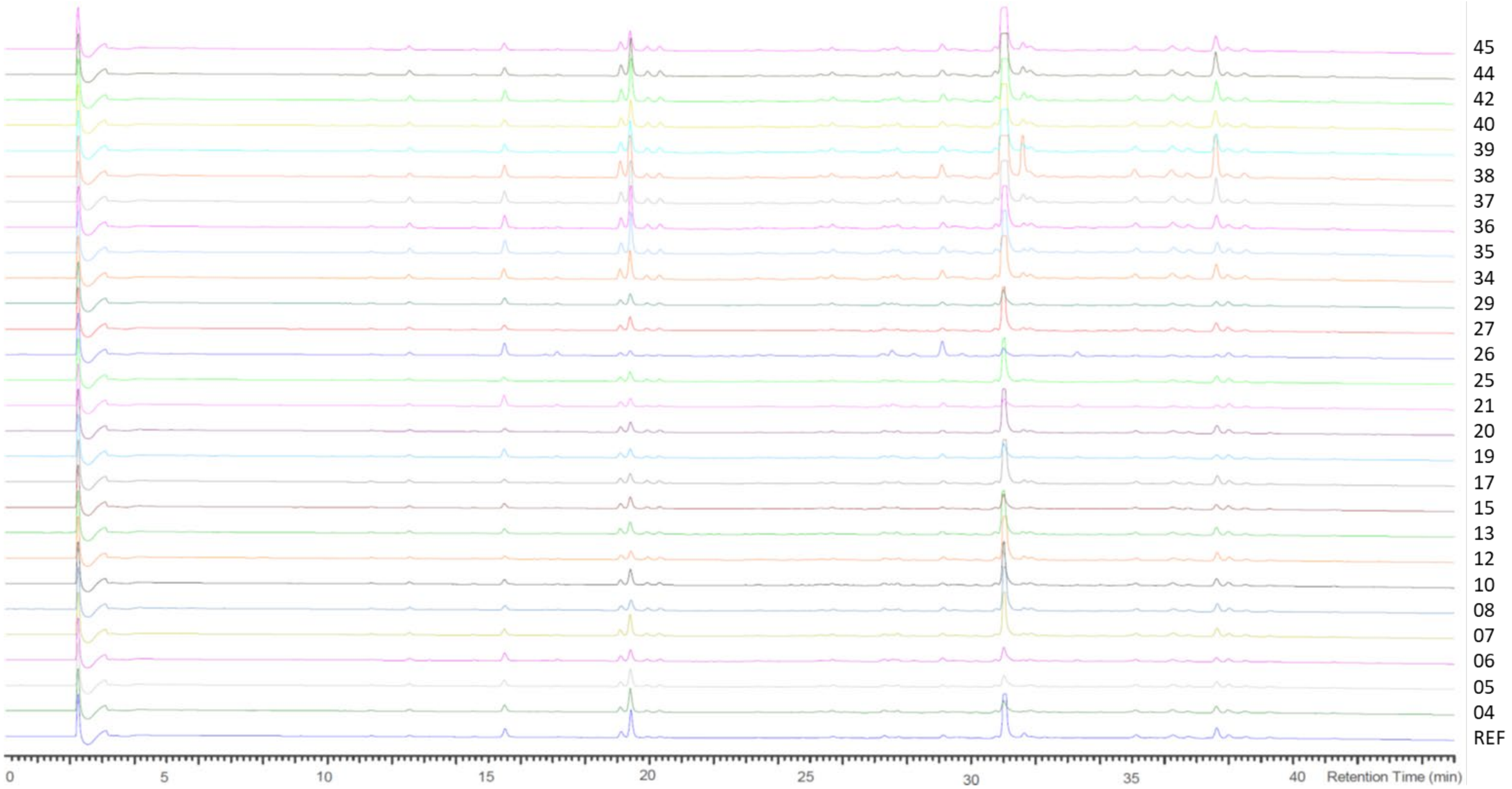

Figure S14. HPLC-PDA analysis at 195 nm of grade C samples. Chromatographic conditions: SunFire C18; 5%-100% MeCN in water (both containing formic acid 0.1%) in 45 min; 0.4 mL/min.

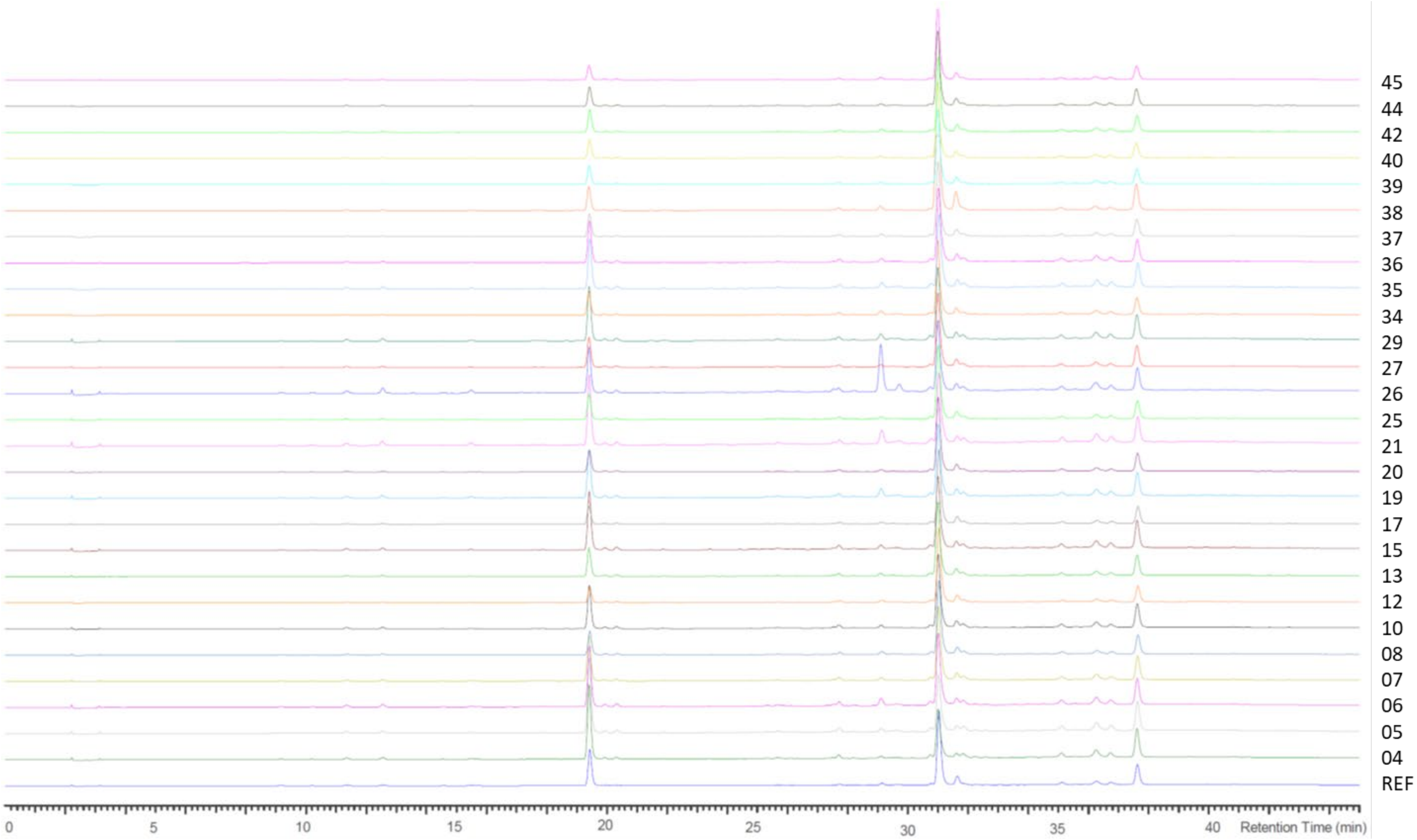

Figure S15. HPLC-PDA analysis at 254 nm of grade C samples. Chromatographic conditions: SunFire C18; 5%-100% MeCN in water (both containing formic acid 0.1%) in 45 min; 0.4 mL/min.

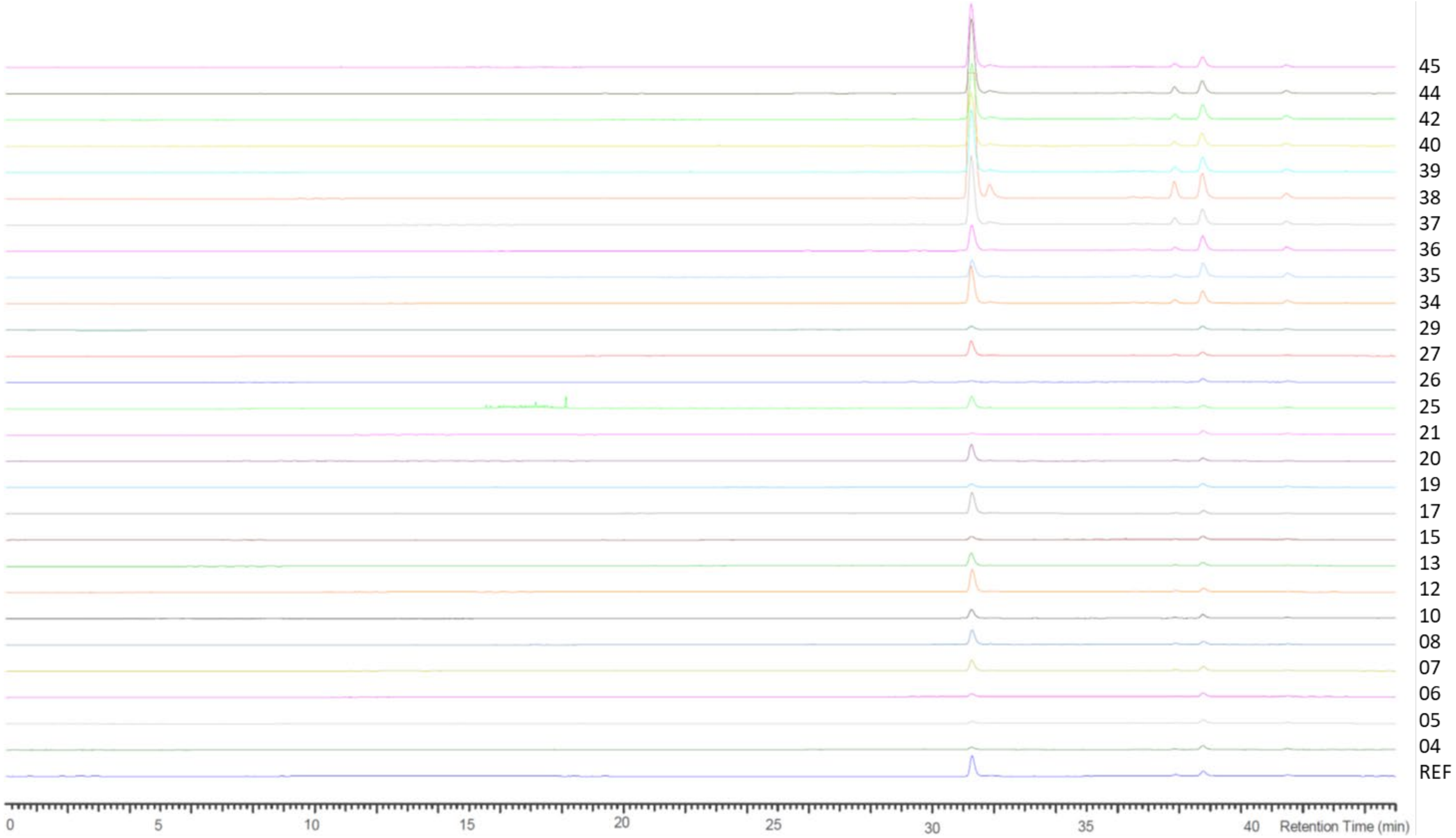

Figure S16. HPLC-ELSD analysis of grade C samples. Chromatographic conditions: SunFire C18; 5%-100% MeCN in water (both containing formic acid 0.1%) in 45 min; 0.4 mL/min.
